# Colorectal Organoids: Models, Imaging, Omics, Therapy, Immunology, and Ethics

**DOI:** 10.3390/cells14060457

**Published:** 2025-03-19

**Authors:** Martina Taglieri, Linda Di Gregorio, Serena Matis, Chiara Rosa Maria Uras, Massimo Ardy, Sara Casati, Monica Marchese, Alessandro Poggi, Lizzia Raffaghello, Roberto Benelli

**Affiliations:** 1 IRCCS Ospedale Policlinico San Martino, 16132 Genoa, Italy; martina.taglieri@hsanmartino.it (M.T.); linda.digregorio@hsanmartino.it (L.D.G.); serena.matis@hsanmartino.it (S.M.); chiararosamaria.uras@hsanmartino.it (C.R.M.U.); massimo.ardy@hsanmartino.it (M.A.); monica.marchese@hsanmartino.it (M.M.); sandropoggi59@hotmail.it (A.P.); lizzia.raffaghello@hsanmartino.it (L.R.); 2 Istituto per l’Endocrinologia e l’Oncologia Sperimentale “Gaetano Salvatore” CNR, 80131 Naples, Italy; sara.casati@cnr.it; 3 Common Service ELSI, BBMRI.it (UNIMIB National Node Headquarter), 20126 Milan, Italy

**Keywords:** PDO, colon–rectum, culture models

## Abstract

Colorectal epithelium was the first long-term 3D organoid culture established in vitro. Identification of the key components essential for the long-term survival of the stem cell niche allowed an indefinite propagation of these cultures and the modulation of their differentiation into various lineages of mature intestinal epithelial cells. While these methods were eventually adapted to establish organoids from different organs, colorectal organoids remain a pioneering model for the development of new applications in health and disease. Several basic and applicative aspects of organoid culture, modeling, monitoring and testing are analyzed in this review. We also tackle the ethical problems of biobanking and distribution of these precious research tools, frequently confined in the laboratory of origin or condemned to destruction at the end of the project.

## 1. Organoids as Models

Human colon tissue culture has a long history [1]. The first colorectal cancer (CRC) cell line (colo205) was established in 1957, and most 2D CRC cell lines grown on plastic have at least 40 years of in vitro culture/selection. Their long history casts some doubt on their use as reliable models. Two-dimensional cancer cell lines are widely used since they are easy to propagate, manipulate, and genetically modify at relatively low costs. However, they do not represent the complexity of the different tumor cell lineages or the tumor microenvironment (TME) in terms of structure, cell–cell interaction, and accessibility to nutrients [2]. The TME contains immune, stromal, and vascular cells, along with specialized epithelia. Moreover, 3D interactions are maintained by non-cellular components, such as the extracellular matrix (ECM) impregnated by different growth factors and cytokines [3,4]. Indeed, historical 2D cell lines are poor models of this complexity and are rarely “normal”, bearing several mutations that enable their persistence in an alien environment [5]. This leads to strong in vitro selection due to low establishment efficiency. However, they may be more suitable for large-scale production of culture byproducts and for cellular biochemistry research and high-throughput genetic perturbation assays. In fact, their monotypic phenotype allows for more consistent and reproducible results [6].

In 1975, Rheinwald and Green established the first method for culturing normal human keratinocytes on mouse fibroblast feeders [7]. However, we had to wait for the 2011 paper by Sato and Clevers to get the first reliable method for long-term culture of normal and pathologic human intestinal epithelium [8]. Key modulations of signaling pathways required for the indefinite expansion of human intestinal organoids included agonism of the beta-catenin (Wnt3A, R-spondin) and Erk1-2 (EGF, SB202190) pathways, and inhibition of BMP (Noggin) and TGFβ (A83-01) signaling. To limit apoptosis during primary culture plating and culture splitting by trypsin digestion, accessory molecules such as the ROCK inhibitor Y27632, gastrin, and nicotinamide were also added. This milestone has revolutionized the culture of most primary epithelial cells in a relatively short period of time, allowing the creation of complex multilineage models from different organs that better reflect the in vivo situation. Colorectal organoids better mimic the natural tissue environment, preserving the different cell populations and differentiation stages found in real tissue (i.e., stem cells, enterocytes, goblet cells, and neuroendocrine cells). Organoid models are best suited for studying human tissue biology and physiology and for clinical applications such as precision and regenerative medicine. On the other hand, they are more difficult and costly to culture and maintain [2] and they are still limited in the representation of the TME [9].

### 1.1. Sources and Propagation of Colorectal Tissue

Once a working in vitro condition for colon epithelial culture was established, the need for sources of primary cell lines increased. Patient-derived primary organoid cultures require ethics committee approval and patient consent, while prospective enrollment can take a long time. Primary cultures can be contaminated by the bacteria living in the intestinal lumen, and cell culture yield is seldom 100%. However, organoids are the most closely matched to their donor, allowing the creation of mini-guts that maintain the genetic characteristics of primary tissues, opening a new perspective for personalized therapies [10].

Colorectal organoids from non-neoplastic pathologies have been successfully cultured using the same conditions suitable for normal mucosa. Dotti et al. established primary organoids from ulcerative colitis (UC) patients [11]. Interestingly, these cultures maintained in vitro the pathological expression signature found in the tissue of origin, including genes related to antimicrobial defense and an altered secretory–absorptive phenotype. Neyazi et al. found that among UC patients, those with primary sclerosing cholangitis showed selective upregulation of the stem cell gene *OLFM4* and downregulation of the R-spondin receptor *LGR5* in primary organoids, a signature previously associated with early stages of neoplastic transformation of the biliary tract [12]. Howell et al. analyzed DNA methylation of intestinal cells from pediatric patients with inflammatory bowel disease (IBD) [13]. They found a stable methylation pattern, distinct from healthy controls, in IBD-derived epithelial cells from the terminal ileum and colon that was partially maintained in ex vivo organoid cultures. Freire et al. established primary organoids from healthy and celiac patients [14]. Exposure to gliadin resulted in increased intestinal permeability and secretion of pro-inflammatory cytokines in celiac organoids, while RNA-seq analysis showed the alteration of genes associated with the intestinal barrier and innate immune response. The Human Individualized Treatment for Cystic Fibrosis (HIT-CF) Europe Organoid Study demonstrated the feasibility of generating a prospective, multicenter collection of organoids from CF patients with a very high success rate (95%) [15], suggesting that this approach could be applied to personalized medicine. According to these examples, a single culture protocol enabled the generation of primary cultures from different pathologies of the digestive tract, filling a previously unmet need for specific models.

On the contrary, the collections of CRC-derived organoids, independently published by Sato and Clevers in 2016 [16,17] showed that most tumor tissues required modified conditions for in vitro proliferation (i.e., hypoxia, SB202190 withdrawal, or dose reduction, etc.). In fact, two modified protocols emerged from these papers, with that from the Clevers study prevailing in most subsequent studies due to its simpler application, despite lower overall success in culture establishment.

**Figure 1:**
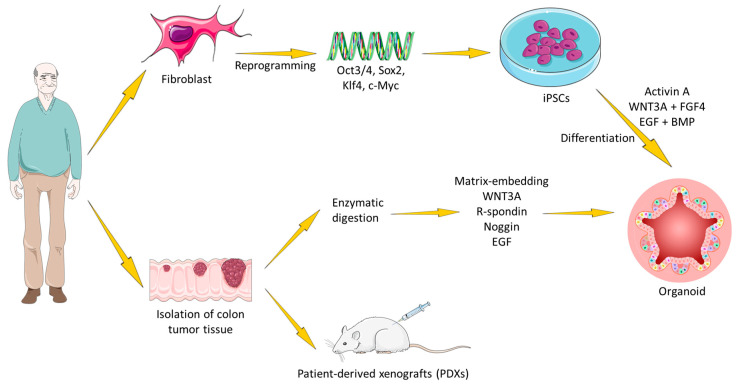
Graphic illustration of organoid derivation models. Upper: Patient-derived fibroblasts can be reprogrammed by transfection of some genes such as Oct3/4, Sox2, Klf4, and cMyc, generating induced pluripotent stem cells (iPSCs). iPSCs can differentiate into epithelial cells in the presence of an appropriate mixture of factors. Lower: Epithelial organoids can be obtained from colorectal carcinoma (CRC) specimens after enzymatic digestion and culturing in extracellular matrix with growth factors. Patient tumor cells can be xeno-transplanted in mice (PDXs: patient-derived xenografts), generating a copy of the original tumor. Although not shown, all these models can be studied by applying OMICS, biochemical, and functional analyses in a culture system with other components of the tumor microenvironment, such as fibroblasts or immune cells.

While all of these patient-based approaches are extremely relevant for pathology-based studies, many basic biology projects would benefit from a readily available model with few ethical and sampling complications. The generation of gastrointestinal (GI) organoids from pluripotent stem cell (PSC) lines, including embryonic and induced PSCs, represents a major advance in modeling human development and disease (Figure 1). Adult human fibroblasts can be de-differentiated into iPSCs by transduction with the “Yamanaka” factors (Oct4, Klf4, Sox2, c-Myc) [18]. Subsequently, iPSCs can be expanded indefinitely and used to generate any tissue, including the gut. Two similar three-step protocols are available for generating colon organoids, differing only in the last step [19]. Both protocols use Activin-A as the first factor to push iPSCs towards endoderm differentiation, followed by Wnt3a + FGF4 to obtain mid-hindgut specification with CDX2 marker expression. One protocol expanded the obtained cystic spheroids using Matrigel embedding and organoid medium (EGF + R-spondin + Noggin) [20]. The other protocol induced a further differentiation step by a short EGF + BMP treatment [21], followed by standard organoid medium subculture. The latter protocol is more effective in inducing colon-specific markers, though it also reduces culture viability, committing many cells to terminal differentiation. As we will report in this review, patient and PSC-derived organoids are not exactly the same. For example, PSC-derived organoids usually show a higher adaptability to in vitro culture and to synthetic matrix embedding, representing a first choice for tissue engineering. The maturation of somatic cell-derived organoids is closer to that of adult tissues compared to PSC-derived organoids, making them more suitable for studying the mechanisms underlying the repair of adult tissues and their response to viral infections [22]. Moreover, the structures derived from normal or diseased epithelial tissues can be expanded in vitro for long periods of time, maintaining genetic stability and making them ideal for the expansion of cells from single individuals or groups of interest and for testing therapies. On the contrary, PSCs are genetically less stable and stop their expansion when they reach terminal differentiation. In addition, they require a more complex culture system, with different factors in the medium that should be adequately combined to allow their growth and differentiation. However, they are useful for development studies where three germ layers are evaluated, or for the generation of organic models with different autologous cell types, such as mesenchymal, epithelial, and endothelial cells [22,23].

Although the focus of this review is organoids, we must also mention patient-derived xenografts (PDXs) as a valuable mouse model for human CRC propagation and testing. While costs, time for tumor establishment and further propagation (6–8 months and 40–50 days, respectively), and engraftment rate (70%) are problematic, PDXs allow epithelial tumor cells to grow along with a murine stroma, creating a niche similar to the original tumor [24]. PDXs maintain the molecular characteristics of the tumor of origin, allowing for more accurate analysis compared to traditional models [25]. PDXs are also useful for assessing drug sensitivity and resistance, facilitating personalized therapy. The creation of PDX models involves transplanting human tumor tissues into immunocompromised mice (engraftment rate: BRG/BRJ  >  NSG  >  NOD/SCID  >  SCID  >  nude) using techniques such as subcutaneous, orthotopic, or intracapsular fat pad implantation to preserve the tumor’s architecture and genetic diversity [26]. Despite their advantages, PDX models require strict quality control and raise double ethical challenges related to the use of human tissues and animal models.

### 1.2. Matrigel vs. Hydrogel

Three-dimensional matrices are used to support the growth, differentiation, and organization of organoids. The most commonly used matrix is the Engelbreth–Holm–Swarm (EHS) mouse sarcoma-derived matrix (known as Matrigel, Geltrex, Cultrex-BME etc.), which provides a three-dimensional environment that mimics the natural ECM essential for cellular support and differentiation. Matrigel is rich in bioactive basement membrane components, such as collagen IV, laminin, and proteoglycans, which promote cell self-organization and the formation of complex, organ-like structures [27]. The role of Matrigel as a critical component in organoid cultures has been widely debated, particularly in contexts requiring reproducibility and clinical applicability. Matrigel exhibits batch-to-batch variability and undefined growth factor composition that need to be reduced/eliminated for organoid culture [27]. Moreover, some batches are contaminated by the mouse lactate dehydrogenase-elevating virus (LDV) that can spread in Matrigel-injected mice [28]. An interesting alternative to the use of Matrigel solid domes has been proposed by Hirokawa et al., who demonstrated that low viscosity Matrigel (5% in organoid medium) enables the culture of normal and cancer colon organoids under floating conditions, improving scalability and accessibility [29].

Recent advancements in synthetic alternatives indicate the successful development of chemically defined and xenogeneic-free matrices. Synthetic scaffolds could allow precise control of mechanical and biochemical properties, facilitating improved cell behavior and reproducibility, though somatic cell-derived organoids are difficult to adapt to artificial scaffolds. In the context of gastrointestinal organoids, Kim et al. demonstrated that decellularized ECM (dECM) derived from gastrointestinal tissues can effectively replace Matrigel [30]. Their findings suggest that organogenesis and functionality of gastric or intestinal organoids are enhanced in these gels compared to Matrigel, particularly for long-term culture and transplantation capabilities. Of course, the use of a human-derived matrix poses new ethical and supply problems. While Matrigel has been a cornerstone in organoid culture, its limitations are increasingly recognized, perhaps the most important being the presence of mouse proteins in immunological models based on human cells, which can activate and expand CD4 T lymphocytes [31]. The transition to alternative matrices not only addresses the issues of variability and undefined composition associated with Matrigel but also aligns with technological advances in organoid research, emphasizing reproducibility, safety, and physiological relevance.

PEG (polyethylene glycol)-based hydrogels can replicate a natural three-dimensional environment that mimics the ECM conditions essential for the proper functioning of intestinal stem cells (ISCs). ISCs respond not only to soluble biochemical signals but also to mechanical and physical stimuli from the matrix, and the ability of hydrogels to mimic these conditions is critical for their controlled expansion and differentiation. The derivatization of synthetic PEG gels with arginyl-glycyl-aspartic acid (RGD) peptide sequence and mouse laminin-111 peptides allows ISCs and mature organoids to survive, while the presence of hydrolytically degradable domains allows organoids to increase in size without being constrained by the matrix [32]. Despite this partial success, this matrix still contains a mouse laminin peptide and was only able to expand mouse organoids, while human colon crypts could survive at most two passages. This is a common problem with most synthetic gels: they usually allow the expansion of mouse somatic tissue or human stem cell-derived organoids [33,34], while human primary organoids derived from somatic tissues do not adapt. There is at least one notable exception to this rule. Hernandez-Gordillo et al. described a PEG-based hydrogel containing synthetic fibronectin (PHSRN-K-RGD) and collagen (GFOGER) peptides, as well as basement membrane and fibronectin-binding peptides, capable of capturing the ECM directly produced by organoids [35]. The GFOGER peptide bound to PEG20 macromers was found to be critical for culturing human organoids, while the mechanical properties of the matrix appeared to be irrelevant. This fully artificial matrix also allowed the expansion of human organoids from single cells, representing an interesting alternative to Matrigel. Another valid alternative is QGel CN99, a synthetic gel with unknown constituents. QGel CN99 was described as completely overlapping Matrigel activity, except for primary culture cell number survival, which was halved [36].

### 1.3. Matrigel vs. Collagen-I

As reported in the previous paragraph, the GFOGER collagen-I peptide maintains the viability of colon organoids in synthetic hydrogels; accordingly, collagen-I could be a suitable matrix for organoids. Although this Matrigel substitute has not been widely reported in the literature, it has shown promising results. Jabaji et al. used pig collagen-I to culture human small intestine-derived organoids along with fibroblasts [37]. Organoids cultured in a fibroblast-derived conditioned medium were expanded to 12 passages in collagen-I and showed an overlapping expression of intestinal epithelial markers (LGR5, Vil1, CDX2, CHGA, and others) with Matrigel-grown controls; only ALPI expression was halved. In the absence of a fibroblast-conditioned medium, organoids showed a rapid degeneration within 2–3 days, despite the presence of most of the supporting factors in standard Sato and Clevers medium (except for A83-01 and SB202190 inhibitors). Jee Hyun et al. tested pig collagen-I dissolved in F12 and polymerized by bicarbonate for culturing mouse organoids from different districts (stomach, small intestines, colon) [38]. Colon organoids showed an overlapping expression of markers (Lgr5, E-cad, Vil1, Muc2, and others) compared to Matrigel-cultured controls. This overlap was not observed for small intestine and stomach organoids. The authors reported that collagen-I also supported the growth of human colon organoids, but the number of samples tested was not reported, and the organoids were cultured for only six passages. Real-time PCR marker comparison showed enrichment in LGR5 and enterocyte-specific markers (Vil1, Lyz), while goblet cell markers (Muc2, Muc5AC) were downregulated. Sachs et al. used floating collagen-I gel disks to obtain the self-assembly of tube-like structures starting from mouse small intestinal crypts [39]. It could be quite interesting to translate this all-mouse model to human organoids as it allows the formation of gut-like structures, with a mature luminal epithelial lining and crypt-like structures invading the matrix. Ramadan et al. used a mouse small intestine model to compare the effects of collagen and laminin exposure on organoids [40]. They found that collagen induced the appearance of a subset of cells with fetal markers, while Matrigel promoted the LGR5+ cell population. Although, the data from these four studies are not fully corroborated, collagen remains an interesting basis for the development of more defined hybrid matrices.

### 1.4. Organ on a Chip

**Figure 2:**
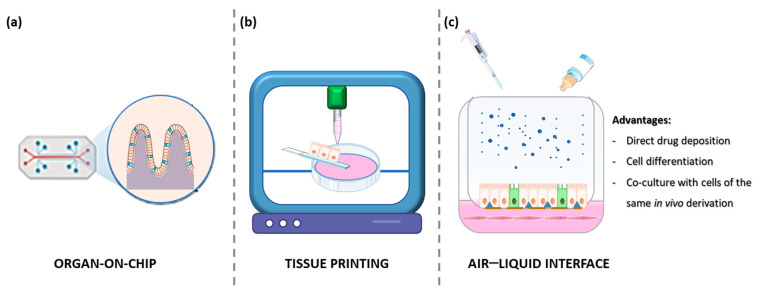
Advanced technologies for the generation of 3D tissue models. (**a**) Organ-on-chip: a system combining cells, microenvironment, and microfluidic tools to reconstruct proliferative and differentiated regions of intestinal epithelium; (**b**) 3D tissue printing: a system that reproduces the anatomy and cellular localization of a fully developed organ for the generation of complex models; (**c**) Air–liquid interface: a system that allows cell growth and differentiation under in vivo-like conditions, with advantages such as direct drug deposition and co-culture with different cell types.

Organs-on-chips (OoCs) are artificial systems containing engineered or natural miniature tissues grown inside microfluidic chips (Figure 2) [41]. The integration of OoC technologies with intestinal organoids has been a significant advance in biomedical research, improving understanding of gastrointestinal physiology and pathophysiology. Several recent studies have highlighted the effectiveness of microfluidic platforms in simulating in vivo environments, improving the relevance of preclinical models and leading to more personalized treatments. Xiang et al. revised the microfluidic systems combined with intestinal organoids derived from iPSCs, embryonic cells, and patient tissue grown on chips, which are able to mimic complex environments essential for the study of diseases such as ulcerative colitis and colon cancer [42]. Zhang et al. created a gut epithelium–microbe–immune (GuMI) OoC to recapitulate the immune response to an oxygen-intolerant bacterium (*Faecalibacterium prausnitzii*) [43]. This short-term (1 week) model contained human intestinal organoids plated on a collagen-coated filter, heterologous antigen-presenting cells (APCs) and naive CD4 T cells (in the lower chamber, below the epithelial monolayer), and F. prausnitzii in the upper, anoxic chamber. By integrating data from GuMI models lacking bacteria, APC, or CD4 T cells, the authors were able to partially dissect the role of each cell population on the overall response. Similar studies on the interactions between microbiome and inflammation could open new avenues for targeted treatments of intestinal dysbiosis, using OoC platforms as the basis for personalized therapeutic strategies. Apostolou et al. developed a colon chip platform based on human colon organoids and microvascular intestinal endothelial cells [44]. Organoid-derived epithelial cells were plated in the upper chamber of the chip treated with a collagen IV + Matrigel + Fibronectin mixture, while endothelial cells were plated in the lower microfluidic chamber. Once the epithelium had organized into a mature, non-permeable monolayer, the lower chamber was used to test the effects of IFNγ or IL22 on epithelial permeability. While IFNγ exerted the intended effects of reorganizing apical junctional complexes and inducing epithelial apoptosis, IL22 acted as a permeability disruptor, despite the protective effects seen in mouse models. These advances are further supported by the work of Palikuqi et al., who introduced innovations in endothelial cell culture by creating functional vascular networks in organoids [45]. These vascular networks are essential for pharmacological testing, allowing better assessment of drug pharmacodynamics in a physiological context. The IFlowPlate OoC platform [46] enables the self-assembly of an organoid with a surrounding and infiltrating vasculature. The perfusable HUVEC bed can be cultured with organoids by mixing 10% Matrigel in a Fibrin gel and using a 50% mixture of organoid and endothelial cell medium. This OoC allowed the culture of vascularized organoids for up to 13 days to study TNFα-induced monocyte adhesion, extravasation, macrophage differentiation, and organoid infiltration. This system allows the study of controlled fluid conditions, enhancing the physiological relevance of the test environment.

A recent OoC model by Mitrofanova et al. was able to generate true human mini-colons using a hydrogel scaffold coated with a collagen–Matrigel (75–25% *v*/*v*) solution [47]. Organoids were plated as a single cell suspension and allowed to recover for two days in complete medium supplemented with a fibroblast-conditioned medium. Subsequently, the components of the medium were switched, giving the full medium without EGF but with NRG1 and A83-01 added only at the base of the artificial crypts. On the other hand, a simplified medium with EGF, noggin, and R-spondin was used for the liquid interface, mimicking the intestinal lumen. This difference created a stem factor gradient that allowed a selective enrichment of LGR5 and proliferating cells at the base of the crypts. Maturation of the mini-colons was established after one week, although the appearance of specialized markers of differentiated enterocytes and goblet cells took 2–3 weeks. Mini-colons recapitulate most physiological and pathological responses and have also been used to establish multicellular cultures. While this model is quite tricky and complex for the average lab, it represents a very advanced OoC for colon tissue mimicry.

### 1.5. Air–Liquid Interface (ALI) Cultures

Current research shows that colon organoid cultures in the air–liquid interface (ALI) effectively recapitulate the in vivo environment (Figure 2). This method supports the faithful representation of stem cell niches, enhances epithelial differentiation, and provides insight into the pathology of gastrointestinal diseases. ALI was one of the first methods to support the long-term propagation of neonatal mouse intestinal tissue. In 2009, Ootani et al. established a mixed epithelial–fibroblast ALI culture in collagen, allowing the persistence of small gut-like structures for 30–150 days [48]. Under these conditions, an R-spondin–Fc chimera increased the efficiency of ALI, allowing the culture of adult mouse tissue for up to 28 days. This study showed that ALI culture not only facilitated organoid growth, but also preserved their differentiation potential, showing the expression of differentiation markers of multiple intestinal epithelial lineages. Dotti et al. showed that in submerged cultures of human colon organoid-derived monolayers, stem/early cell markers were predominant, whereas ALI culture allowed a progressive enrichment of populations differentiated towards absorptive and secretory lineages [49]. Similar observations were reported by Sabapaty et al., observing a better expression of late differentiation markers in ALI cultures compared to 3D matrix-embedded, submerged cultures [50]. Indeed, the observation of increased differentiation of colon tissue in ALI cultures is reported by most authors.

Wang et al. provided important information on the cycles of injury and repair in a mouse colitis model based on ALI-cultured intestinal stem cells and reported the identification of a regenerative Hopx+ stem cell population [51]. Mouse intestinal organoid-derived cells were plated on a Transwell filter coated with 10% Matrigel and cultured as monolayers, thus not representing the real 3D organization of intestinal crypts. This ALI model was able to replicate the in vivo observations where the Hopx+ population was responsible for epithelial regeneration, while LGR5+ cells were poorly represented. Li et al. contributed to understanding the oncogenic transformation of primary mouse organoid cultures [52]. While KRAS G12D mutation and P53 loss were sufficient to transform pancreatic and gastric organoids into carcinoma-like tumors, colon organoids required four events (Apc, p53, Kras, and Smad4 mutations) to acquire invasive properties and a carcinoma-like appearance. The latter finding was replicated one year later in human 3D organoid cultures (without ALI) [53]. Liu et al. created a mucosoid ALI model of human gastric intestinal metaplasia by plating 2D cells as a monolayer on a collagen gel [54]. This model was able to reflect the expression of markers found in the primary tumor lesions and to identify potential new markers to aid in the diagnosis of this metaplasia.

ALI technology has also been applied to mixed co-cultures of intestinal bacteria with primary human colonic epithelium. Kim et al. created an ALI designed to mimic the anaerobic conditions of the colon, where most intestinal bacteria thrive as obligate anaerobes [55]. This ALI model accumulated a functional 100 µm thick layer of mucus acting as a barrier between the intestinal epithelium and bacteria, allowing the co-culture of Lactobacillus rhamnosus and Anaerobutyricum hallii on the colon epithelial layer without any damage to the epithelium, resembling the physiology of the in vivo situation. Altogether, these examples suggest that while ALI frequently represents a back-step to a 2D-like model, it can better represent the outer surface of mature colon epithelial cells, an ideal approach for barrier integrity and microbiota-related studies.

### 1.6. Tissue Printing

Tissue printing’s primary objective is to replicate the intricate anatomy of a fully developed organ, accurately depicting the expected location and function of the majority of cell populations (Figure 2). To achieve this goal, both matrix and cells can be used as ink for 3D printing. Four bioprinting techniques are widely used: inkjet-based, laser-assisted, extrusion-based, or photo-curing, each with its own advantages and disadvantages (see [56] for a review). Brassard et al. used Matrigel, collagen, or Matrigel + collagen premade gels, injecting highly concentrated cell suspensions as an ink [57]. These authors demonstrated that the plating method was crucial for enabling the effective self-assembly of cells into mature structures. Mouse organoid-derived cell suspensions demonstrated enhanced performance when injected as a linear array, while mouse mesenchymal cells exhibited optimal functionality when plated in small aggregates dispersed at regular intervals. The printing of both cell populations in the same gel allowed for their rapid interaction and a faster organization of intestinal epithelial cells into a small gut-like structure compared to epithelial cells plated alone. Han et al. used a decellularized matrix derived from porcine colon as an ink to print a perfusable tubular structure [58]. The major component of this matrix (73%) was collagen type VI. The decellularized matrix allowed the enrichment of SOX9 and LGR5-positive stem cells in long-term organoid cultures compared to Matrigel controls. The differentiation markers Vil1, MUC2, and ALPI were also increased. When the matrix was mixed with colon cells, this ink could be used to print tubes that would eventually develop into small intestines, with mature cells facing the lumen and stem/proliferating cells forming small crypts in the matrix. While most of the experiments were based on the CaCo2 tumor cell line, they were also partially replicated using primary human small intestine organoids.

Although, the reconstruction of healthy tissue is the primary endpoint for regenerative medicine and physiology studies, tissue printing was also applied to CRC modeling. Many studies rely on historical CRC cell lines, with some interesting confirmations based on patient-derived cells. Cadamuro et al. prepared a bioink based on hyaluronic acid and gelatin enriched with one of three signaling glycans (3′-sialylgalactose, 6′-sialylgalactose, or 2′-fucosylgalactose) [59]. Sialylation and fucosylation induced different modulations of the proteome in primary HT29 tumoroids. In primary human CRC tumoroids, 6′-Sialylgalactose was able to induce the selective expansion of LGR5 and CD133 stem cells, identifying a key factor involved in tumor stem cell proliferation. Sun et al. directly bio-printed primary patient-derived CRC cells from surgical specimens using a gelatin–alginate bioink [60]. This direct printing allowed immediate, short-term establishment of 37/40 primary cultures and 29/31 metastases; the parallel attempt to establish organoid cultures resulted in seven and eight proliferating patient-derived organoids (PDOs), respectively. The 85% of bio-printed primary CRCs and all metastases were viable for about two weeks, allowing immediate drug testing with first-line drugs (5-FU, CPT-11, and oxaliplatin). Although the drugs were only tested individually on each CRC and mCRC printed sample, the test showed correspondence with patient responses. In fact, samples resistant to all drugs corresponded to patient progression, while samples sensitive to all drugs showed the best patient responses. Intermediate overlap was also observed. Although this model allows only short-term drug testing, it seems to be quite valuable for the immediate postoperative evaluation of most samples. Its integration into a clinical routine could be facilitated, saving time and resources required for organoid or PDX establishment.

### 1.7. Multicellular Assembloids

Throughout the chapters in this review, we will frequently highlight the growing need to reconstruct multicellular models capable of mimicking the original tissue. This goal is not easy to achieve because the culture conditions that allow epithelial organoid growth and survival are not tolerated by other TME components. For example, fibroblasts and leukocytes require FCS to grow and can be negatively affected by the activity of some organoid medium components (e.g., nicotinamide for lymphocytes and TGFβ inhibitors for fibroblasts). In this and the following paragraph, we will briefly review some successful conditions used to achieve viable multicellular (and multi-organism) co-cultures.

**Figure 3:**
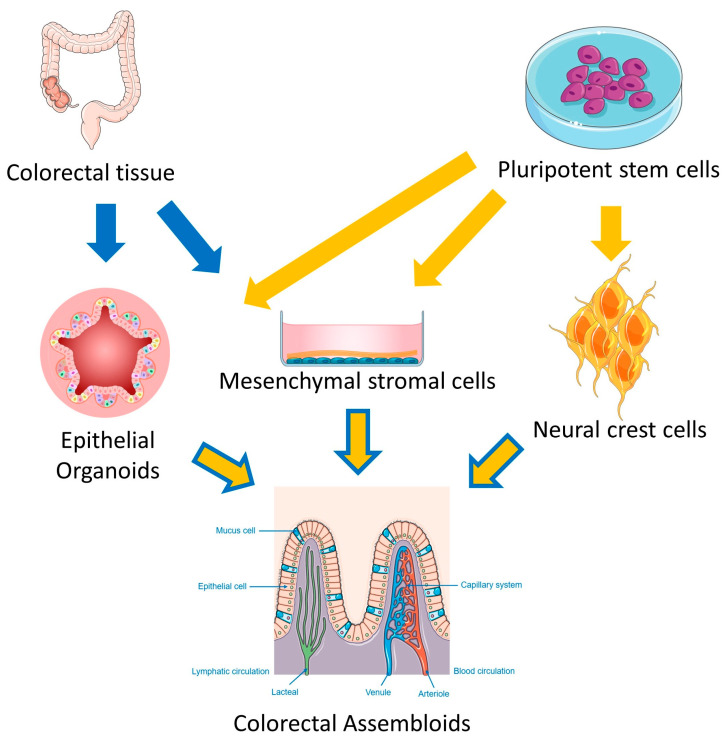
Scheme of gut assembloids generation. In summary, these complex gastro-intestinal models are developed from intestinal stem cells isolated from colon biopsies or pluripotent stem cells derived from human intestinal cells. After the differentiation in neural crest cells, gut spheroids, and mesenchymal cells, the three different subpopulations obtained are combined to form the final assembloids.

Various models of assembloids (Figure 3) have been generated to study the interaction between different cell types in colorectal tumors. In particular, many studies have established these models using primary adult stem cell-derived organoids or by iPSCs differentiation [61,62]. A crucial step is the embedding of the cells in a suitable matrix, mainly Matrigel, to allow the acquisition of a 3D structure [61]. This matrix resembles the extracellular environment of the basement membrane, thanks to the presence of laminin and collagen IV, which are fundamental for the induction of endothelial cell interactions and for the migration of immune cells within the epithelial layer, as shown by some studies [46,63]. Lin et al. assembled mouse intestinal epithelial and stromal cells to generate intestinal-like assembloids in vitro [64]. The authors found that the simple mixture of these cells did not organize coherently, and fibroblasts escaped from the matrix adhering to the plates. Therefore, murine intestinal organoids were grown under standard conditions and, when well developed, each organoid was placed directly in the center of a drop of Matrigel containing a stromal cell suspension. Culturing these assembloids in standard organoid medium on an orbital shaker allowed them to organize into mature intestine-like tissue within 4 days. Indeed, crypts with basal proliferating cells and apical multilineage differentiated cells were observed. Moreover, fibroblast populations were organized to support epithelial homeostasis, with Rspo-3-producing cells at the base of the crypt and BMP-producing cells in the luminal part. The organization of epithelial and stromal cells in this model was striking, as it completely mirrored the in vivo situation [64].

To study intestinal crypt maturation, other groups have introduced enteric nervous cells into intestinal assembloids. Workman et al. organized the enteric nervous system (ENS) in vitro from human EC or iPSC-derived multipotent neural crest cells (NCCs) [62]. iPSCs were differentiated into intestinal organoids using a standard protocol (Activin -> FGF4 + CH99021 -> EGF). NCCs were derived from iPSCs using retinoic acid followed by FGF2 + EGF + insulin. Both cell populations were assembled into 3D matrix domes without trypsinization and were allowed to mature for 28 days. Epithelial cells were found surrounded by βIII-tubulin+ neurons and S100β+ glia embedded in the mesenchyme. The organization of epithelium, mesenchyme, and neuroglial cells was reminiscent of the 11.5-day embryonic mouse intestine, suggesting regulation of ENS cells by the organoids. In support of this hypothesis, intestinal organoids expressed both glial cell line-derived neurotrophic factor and endothelin-3, two chemoattractants that are fundamental in regulating NCC migration into the intestinal mesenchyme.

A paper by Eicher et al. focused on studying the interaction between the nervous system and the intestinal epithelium in a 3D model [65]. This multicellular gastrointestinal system was based on foregut iPSC-derived spheroids supplemented with enteric neuroglial cells (ENCs). ENCs were generated from human PSCs cultured in neural induction medium to form neurospheres grown on fibronectin as described by Workman [62]. Mesenchymal cells were derived from hPSCs using the Activin A, BMP4 + CHIR99021, and FGF2 + PIK90 (PI3K inhibitor) differentiation protocol. The aggregation of neuroglial and mesenchymal cells with the spheroids by slow centrifugation and matrix embedding allowed the generation of human antral and fundic gastric tissues. These tissues contained differentiated glands surrounded by smooth muscle layers characterized by functional enteric neurons that regulated the contraction of the engineered antral tissue. The culture medium was a standard organoid medium supplemented with EGF only. The addition of mesenchymal cells along with the ENCs was critical for the generation of functional assembloids. The mesenchymal cells promoted the growth of the intestinal tissue, while the ENCs, after transplantation into mice with mesenchymal cells and the human gastric organoids, contributed to the formation of various layers similar to those surrounding the human stomach in vivo [65].

The development of three-dimensional models composed of intestinal organoids derived from tumor fragments combined with T lymphocytes and fibroblasts has allowed the short-term evaluation of immunotherapy approaches based on the use of bispecific antibodies (TCB), such as cibisatamab (CEA-TCB), and a new CEACAM5-TCB antibody, in combination with a fibroblast agonist FAP-4-1BBL antibody [66]. In this model, T lymphocytes, fibroblasts, and whole CRC organoids were assembled in a Matrigel/collagen I mixture and plated in a microscopy perfusion chamber. The medium used during the 12 h assay was not reported. This model demonstrated more efficient tumor cell killing mediated by CEACAM5-TCB, as the CEACAM5 antigen is more abundant in all CRC cells tested. The response to CEA-TCB depended on the level of surface CEA expression, resulting in a patient-dependent response. The costimulatory effect of anti-FAP-4-1BBL on fibroblasts was evident, enhancing both IFNγ release and tumor cell killing efficiency.

### 1.8. Multi-Organism Systems

The increasing need to understand the interactions between human cells and other species, mainly bacteria, has necessitated the development of more complex systems that could mimic the positive or negative interactions between them, particularly in the case of the intestine where studies have been conducted to evaluate the effect of microbiota on healthy cells and infectious bacterial processes.

A work by Sasaki et al. has illustrated a novel system of scalable co-culture of human anaerobes and colonic epithelium, based on the connection of two chambers [67]. The apical one was characterized by an anaerobic environment due to the presence of an anaerobic gas and a butyl rubber plug to seal the chamber. It allowed the growth of bacteria normally found in the intestine. The basal chamber was maintained under normoxic conditions and was defined by intestinal epithelial cells consisting of actively proliferating cells surrounded by more mature cells. The epithelial cell layer showed an adequate oxygen supply along with intact cell polarity, stem cell hierarchy, and mucin layer. Among the bacterial species introduced into the epithelium, *Bacillus adolescentis* showed the strongest adherence to the human cells. Its growth was more pronounced in the complete cell medium than in the epithelial culture supernatant since the glucose levels essential for its proliferation are generally maintained adequate by the intestinal epithelia. In addition, the intestinal epithelia show an increased expression of stemness markers after interaction with bacteria, indicating the activation of intestinal cells by live microbiota [67].

Recent studies suggest that environmental factors are key drivers of CRC and that the colonic microbiota plays a critical role in both its initiation and progression [68,69]. In addition to microbial factors that induce inflammation and contribute to carcinogenesis by stimulating oxidative stress or altering the stem cell niche, certain gut-dwelling bacteria appear to directly affect the genomic integrity of epithelial cells through genotoxins. In this context, colibactin is a prominent genotoxin synthesized by a set of enzymes encoded by the *E. coli* pks island [70]. The latter is responsible for alkylating DNA at adenine residues and inducing double-strand breaks in cultured cells [71,72].To study genomic instability induced by pks+ *E. coli* in vitro, infections have been performed only on transformed or immortalized cell lines. It remains unclear whether healthy primary colonic epithelial cells respond to infection in the same way. In this regard, a recent study focused on the interactions of bacterial strains of *E. coli* with pluripotent cell-derived intestinal organoids [73]. The use of mucosoids, which are polarized epithelial monolayers grown in an air-liquid interface, was essential because they represent an intestinal environment that closely resembles in vivo conditions. The epithelial cells, organized in columns, expressed on their surface markers such as β-catenin and MUC-2, maintaining their characteristics after the generation of 3D tumor models. In addition, they were highly polarized by the expression of E-cadherin in the basolateral region and the eccentric localization of the nucleus. Exposure of mucosoids to colibactin-producing *E. coli* resulted in an increased mutational burden, including chromosomal aberrations typical of genomic instability. Colibactin also caused mucosoid cultures to become Wnt-independent for their propagation, an effect probably driven by several mutations affecting genes related to p53 signaling, including miR-34a. The use of mucosoids instead of organoids was crucial to observe colibactin-induced changes such as megalocytosis (cell enlargement) and the formation of multinucleated cells [73].

To study the interactions between bacteria and the human intestine during infection and to demonstrate the direct role of *E. coli* in the induction of oncogenic mutations, human intestinal organoids were derived from surgically resected CRC tissue or endoscopic samples from patients with Barrett’s esophagus. These organoids were exposed to genotoxic pks+ island *E. coli* isolated from CRC in a culture system containing a soluble form of the basement membrane and various growth factors. Whole genome sequencing of clonal organoids before and after this exposure revealed a distinct mutational signature that was absent in organoids injected with isogenic pks mutant bacteria [74].

All these studies highlight the promising role of a complex multicellular system in predicting the positive or negative effects of bacterial action on specific human tissues and emphasize the need for appropriate support, especially matrix and growth factors, to create in vivo-like conditions.

## 2. Organoids Imaging and Image Analysis

**Figure 4:**
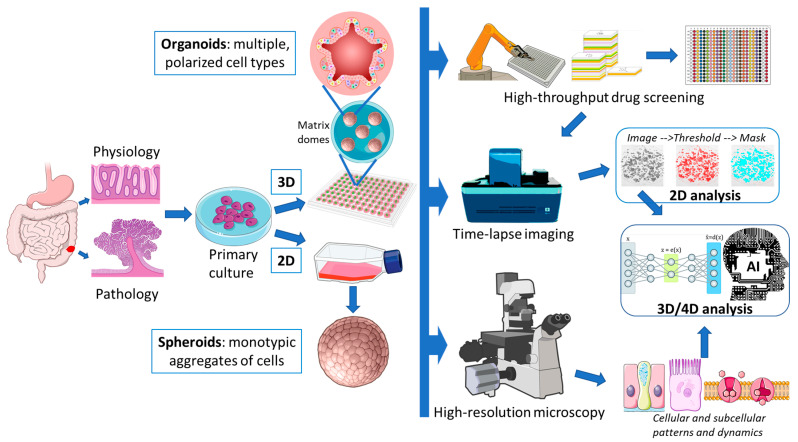
Colorectal primary culture models and their image analysis frameworks. Colon cells can be derived from healthy and/or diseased portions and cultured under appropriate conditions to obtain primary conventional (2D) or three-dimensional (3D) primary cell lines. Three-dimensional organoids are characterized by polarization of multiple epithelial cell types inside the matrix domes. On the other hand, culturing primary cell lines can generate monotypic aggregates of epithelial cells with a typical spherical shape termed spheroid. The sensitivity of organoids and/or spheroids can be assessed by high-throughput drug screening or time-lapse imaging and analysis by conventional software or advanced software based on artificial intelligence (AI). To study the features of cells present in the organoids/spheroids in great detail, advanced microscopy (confocal microscopy, light-sheet microscopy, and super-resolution microscopy) and image analysis should be applied.

The 3D culture of colorectal organoids could be quite difficult for a beginner. This is only the first step in a long journey to experimental results. In fact, omics and flow cytometry-based approaches could be easier than any image analysis of these complex and bulky structures (a summary of the techniques described in this paragraph is shown in Figure 4). Most studies still use 2D imaging of fixed and sectioned specimens or limited confocal Z-stacks. This traditional approach is always a good starting point, although time-lapse monitoring of multiple live 3D structures would be a desirable upgrade. In this chapter, we will review various approaches to this problem and propose new protocols/strategies.

The more practical method for imaging multiple organoids is based on live scanners such as Incucyte, CellCyteX, BioTek, JuLI-Stage, and others. These scanners allow an average lab to monitor organoid cultures by detecting both brightfield and fluorescent probe signals on the same sample. This approach is quite simple, and most scanners are equipped with analysis software that can convert images into data at a glance, allowing real-time evaluation of results [75,76]. Monitoring the growth of a large number of heterogeneous organoids with live scanners can reduce the background noise; however, it is usually limited to a simplified analysis of the area occupied by the organoids in the field of view since organoids are analyzed as 2D objects. Most information about the state of the organoids (i.e., overlapping organoids at different Z, drug-induced changes in morphology, stem vs. differentiated 3D organization) is usually not discriminated.

Artificial intelligence (AI) could be a good tool for dissecting these differences, allowing different data to be collected from the same images. Bian et al. created a high-throughput organoid dataset by synthesizing 75 high-resolution images consisting of 16 microscopic fields with 30 Z-stacks every 24 h for 8 days [77]. These images were used to perform two tasks—deep learning (DL) identification of each organoid and its tracking over time. Qualitative identification required downsampling and cropping of individual organoids to reduce computational memory consumption; once identified, the second algorithm was able to correctly track each of them along different full-size images. Although this approach could monitor every single organoid plated in a multi-well plate, its application is far from the routine of the average scientist. Therefore, alternative simplified approaches using 2D images at normal resolution have been developed. Gritti et al. developed the machine learning algorithm MOrgAna, distributed as a Python package (3.7-3.9 versions-compatible), which is able to segment organoids [78]. MOrgAna can be used without any programming experience through a graphical user interface (although installation requires some skills; download link: https://github.com/LabTrivedi/MOrgAna.git accessed on 13 March 2025). It analyzes both bright field and fluorescent features to dissect morphological information of single organoids. While the program has a default preset of pixel features that usually work on most images, it can be finely tuned by the user for more precision in different organoid models. Its comparison with other algorithms (CellProfiler and OrganoSeg) showed superior performances in segmentation and run time. DeepOrganoid is an alternative DL algorithm developed by Powell et al. that works with brightfield images (made of 10 Z-stacks focus interpolations) and is finalized to identify organoid viability [79]. To enable the algorithm to detect viable cells, a cell-titer bioluminescence signal (ATP-dependent) was detected on the same images. The model was trained on nine CRC organoid cultures and validated on HCT116 spheroids treated with different drugs, showing reproducible segmentations (the free Python 3.6 code is available at the link: https://github.com/ReidTPowell/deepOrganoid accessed on 13 March 2025). OrgaExtractor is a more recent U-net-based algorithm developed by Park et al. using human normal colon organoids [80]. The final output of OrgaExtractor is a contour image that provides measurements of the area, perimeter, circularity, and solidity of each organoid (link to the package: https://github.com/tpark16/orgaextractor accessed on 13 March 2025). The shape and size of the organoids detected by this algorithm are proportional to the number of cells forming the organoids, allowing real-time growth quantification. OrganoID, developed by Matthews et al. using U-Net, tracks individual organoids in brightfield images [81]. OrganoID is a notable improvement over other algorithms because it was trained on pancreatic cancer organoids and validated on lung, colon, and adenoid cystic carcinoma organoids with unmodified parameters. This broad applicability suggests a possible translation to different models, as a pan-organoid quantification tool (download at https://github.com/jono-m/OrganoID accessed on 13 March 2025). The main limitation described by the developers is the inability of OrganoID to distinguish organoids overlapping on the same focal plane. D-CryptO is an algorithm developed by Adbul et al. to discriminate wellness/stemness of organoids by detecting budding and opacity [82]. While this capability is of great interest and allows the detection of important parameters on single segmented organoids, the accuracy of budding detection is suboptimal (70%) in the presence of overlapping objects. OrgaSegment is a MASK-RCNN-based DL algorithm specifically developed by Lefferts et al. to evaluate organoid swelling [83]. Working with bright field images, OrgaSegment identifies objects with different shapes and has been applied to the study of the cystic fibrosis (CF) transmembrane conductance regulator (CFTR) ion channel. Intestinal organoids from CF patients have previously been shown to be optimal tools for discriminating residual CFTR activity in the presence of inactivating mutations [84]. The comparison of healthy and CF organoids is particularly efficient upon forskolin stimulation, as this cAMP activator causes rapid swelling of controls. OrgaSegment was shown to be able to discriminate the activity of drugs that rescue CFTR activity. This algorithm is freely available (https://github.com/Living-Technologies/OrgaSegment accessed on 13 March 2025) but requires powerful hardware to work. Since OrgaSegment was specifically trained on intestinal organoids, it could provide a ready-to-use platform for specific studies investigating cAMP activity. While these examples are just a few of the many AI algorithms developed in recent years, their primary application is almost always the accurate segmentation of organoids and the evaluation of their shape in bright field images. In fact, advances in microscopy techniques have made it easier to image details in individual organoids at the cellular and subcellular levels, despite their dimensions, which limit antibody penetration and cause light scattering.

Tissue clearing techniques have been developed to analyze large 3D samples (e.g., whole mouse embryo brain) while preserving their original structure, and could be transferred to large organoid imaging. The CLARITY protocol and its extensions are based on perfusing tissues with acrylamide, bisacrylamide, and formaldehyde to form a tissue–hydrogel hybrid [85]. Once formed, this hybrid is stable and is followed by complete removal of lipids, improving permeability and transparency. CLARITY is a time-consuming protocol, so alternatives such as SeeDB may be preferred. According to SeeDB, PAF-fixed tissues are permeated with a saturated fructose–water solution containing α-thioglycerol as a reducing agent [86]. Fructose has a refractive index similar to that of lipids, so there is no need to remove them. SeeDB does not quench most fluorescent proteins, and lipid-based fluorescent trackers are retained in labeled cells. The combination of CLARITY and SeeDB, applied to super-resolution stimulated emission depletion microscopy, allowed nanoscale resolution of proteins [87]. Edwards et al. proposed expansion microscopy as an interesting extension of these clearing protocols, specifically applied to spheroids and organoids [88]. Expansion microscopy relies on the formation of a tissue–hydrogel hybrid, such as in CLARITY, followed by volumetric expansion. The result is a highly transparent, porous tissue. These properties allow for deep and uniform antibody penetration and very low light scatter. Expansion microscopy allows high-resolution imaging of entire structures without the need for sectioning. A simpler alternative with good performance has been described by Dekker et al. [89]. This protocol, which uses 4% PAF fixation and fructose–glycerol clearing, allows imaging of whole 3D immunolabeled organoids while preserving the fluorescence of reporter genes. This technique has been tested on organoids of different sizes and origins and has been adapted to different microscopy methods (confocal, super resolution, and light sheet). Importantly, the organoids can be stored in the clearing solution at −20 °C for months, and the clearing step can be reversed and new antibodies added at any time.

The next step in imaging is to monitor the organoids dynamically at high resolution. This involves the acquisition of multiple fluorescence images in time lapse (without quenching) to follow the fate of different organoids and their constituent cells. Light-sheet microscopy is the best choice for imaging large volumes, as the planar excitation of the sample strongly limits fluorescence quenching and photodamage of living cells, allowing multiple images to be collected [90]. Eismann et al. developed a high-throughput screening workflow for the automated evaluation of mitotic phenotypes in 3D MCF10A-derived breast cancer spheroids [91]. Images collected by light-sheet microscopy for 24 h at 5 min intervals were processed by an in-house-developed image processing tool. The convolutional neural network classifier showed 96% accuracy in identifying the four major cell cycle stages (prophase, anaphase, metaphase, interphase). Hof et al. used light-sheet microscopy to live-monitor a cholangiocarcinoma organoid culture expressing fluorescent markers for 7 days at single organoid or single cell resolution [92]. Each routine allowed real-time imaging of up to 120 organoids and their analysis through a proprietary pipeline. This approach identifies different growth behaviors and can quantify the heterogeneity of organoid cultures. De Medeiros et al. reported a powerful analysis of intestinal organoids using a multiscale, light-sheet-based framework [93]. Murine small intestine organoids were transduced to express H2B-mCherry (nucleus) and mem9-GFP (cell membrane) fluorescent tags for live fluorescence imaging. Images were acquired every 10 min for 5 days (150–200 μm volume, 2 μm Z-stacks, 100 ms exposure). This framework allowed the reconstruction of the development of 3D digital organoids, tracing the fate of individual cell populations by their fluorescent identification at the end of the assay. LSTree is a freely available software package that consolidates these tools into a unified framework (link https://zenodo.org/records/6826915 accessed on 13 March 2025). A deeper, 4D view of stem cell-derived intestinal organoids was developed by Schöneberg et al. using a lattice light-sheet microscope with adaptive optics [94]. Organoids were labeled with Clathrin-TagRFP and Dynamin2-eGFP and the dynamics of these markers were monitored at a frame/channel frequency of 2.85 s in a volume of 68 × 58 × 40 µm for 400 s, allowing the generation of a microscale movie containing 36 different cells. The huge amount of data could not be processed by conventional software, so a custom framework (pyLattice link: https://github.com/JohSchoeneberg/pyLattice accessed on 13 March 2025) had to be developed. This framework was used to track individual clathrin-mediated endocytosis events, revealing apical, basal, and lateral membrane dynamics in a human polarized live epithelial cell.

According to these examples, high-resolution imaging allows deep insight into organoid dynamics and usually requires high computational resources, specific machines, and technical skills. Despite the common use of several freeware frameworks for analysis, these techniques are rarely replicated outside the originating lab due to the different organoid models and tagging approaches, reducing convergence towards a single standard.

A completely different approach is indicated for high-throughput screening (HTS) of drug libraries, where hundreds or thousands of compounds need to be tested rapidly and reproducibly. In this setting, automated systems that detect a single surrogate marker in 384- or 1536-well formats are usually preferred. Several authors have described validated methods for achieving these endpoints [95,96,97,98]. Most of these studies show common features indicating an increasing convergence towards standardization: (a) robotic plating of organoids in microwells seems to be a must, allowing one to reduce the number of replicates while increasing the reproducibility of the assay; (b) staurosporine-induced cytotoxicity is the preferred positive control for killing, allowing inter-organoid comparisons between different donors/patients; (c) bright-field imaging is usually compared to bioluminescent quantification of ATP consumption. While the latter is considered the gold standard in toxicity assays, HTS imaging techniques are surpassing its efficiency, allowing the extraction of multiple information from the same image, as discussed above. The potential limitations of HTS approaches are (a) the preference for short-term assays capable of monitoring acute toxicity but potentially underestimating biochemical resistance mechanisms; (b) the testing of drugs on small-sized, newly plated organoids, whereas the tumor of origin would be best represented by large-sized and fully developed organoids; (c) the underestimation of the biological activity of constituents of the organoid medium in contrast to drug efficacy; (d) a possible reduced buffering of pH in small medium volumes, causing a biologically relevant acid shift that induces hypoxia-driven responses.

## 3. Omics of Colorectal Organoids

Healthy and patient-derived colorectal organoids faithfully recapitulate key features of the colorectal epithelium, including tissue architecture, genetic profile and physiological functions [99,100,101,102]. These features make colorectal organoids a reliable and versatile model to study normal intestinal biology, disease mechanisms and drug screening.

The application of omics technologies, including genomics, epigenomics, transcriptomics, proteomics, and metabolomics, to tissue organoids is a relatively new approach that offers the opportunity to study tissue-specific biology in a more physiologically relevant context. This is particularly impactful for colorectal organoids, where multi-omics can explore the complexity of interactions in both healthy and disease states. In particular, omics of colorectal organoids derived from healthy tissue provides a powerful approach to understand normal cellular functions and the molecular processes involved in tissue homeostasis. Furthermore, omics analysis of CRC PDOs enables the identification of specific biomarkers that can be used for diagnosis, prognosis, or prediction of treatment response. An important aspect of molecular analysis using omics technologies, such as genomics, transcriptomics, and proteomics, in PDOs is the identification of specific molecular signatures that reflect disease states and therapeutic outcomes.

Omics-based analysis of organoid models can be considered a revolutionary approach for several reasons. It provides detailed information across multiple molecular levels, providing a holistic view of cellular processes. It can process data from large numbers of samples, allowing robust comparative studies. By combining data from different omics layers, omics technologies enable the study of complex interactions between genes, proteins, and metabolites providing a deeper understanding of key molecular mechanisms underlying colorectal diseases and novel therapeutic targets [103,104].

This chapter provides an overview of different omics approaches, including genomics, epigenomics, transcriptomics, proteomics and metabolomics, applied to colorectal organoids. Table 1 summarizes the major omics analyses performed on both healthy and CRC-derived PDOs, highlighting the techniques used and the key findings from these studies.

### 3.1. Genomics

Next generation exome or whole genome sequencing of colorectal organoids derived from neoplastic/inflammatory and adjacent normal tissues has been instrumental in identifying somatic mutations, including mutation burden and signatures, copy number variations, and chromosomal rearrangements. Most importantly, several studies have consistently found that organoids are a reproducible model of the original parental tissue. In fact, they faithfully replicate the genetic characteristics of the tissue from which they were derived, making them a reliable model for studying tissue-specific genetic landscapes [105,129,130]. This has been particularly well demonstrated in organoids derived from colorectal cancer (CRC) patients, where *APC*, *KRAS*, *TP53,* and *SMAD4* mutations have been validated as key drivers of colorectal tumorigenesis [17,101,106,107,131]. Similarly, genetic profiling of a comprehensive library of colon tumors, including benign adenomas and different histologic subtypes of adenocarcinoma (low/high grade, mucinous, and neuroendocrine), identified distinct mutational patterns across different subtypes, with high concordance between the CRC organoids and the original tumors [16,17]. For example, *APC* mutations were observed in all adenomas and most CRCs, whereas *TP53* mutations were present in the majority of CRC organoids but were rare in organoids from benign lesions. Comparative genomic hybridization/single nucleotide polymorphism (SNP) microarray analysis of the above library revealed that all benign organoids form adenomas were diploid with few chromosomal alterations. In contrast, most of the adenocarcinoma-derived organoids showed various chromosomal alterations such as gains of 13q and 20p and losses of 18p and 17p.

A key finding of whole-genome sequencing analysis is the identification of mutational signatures based on the sequence context of somatic single nucleotide variants, which can have endogenous and exogenous origins [129]. Signatures prominently present in CRC are associated with DNA repair deficiencies, namely mismatch repair (MMR) leading to microsatellite instability, defects in base excision repair due to *MUTYH* or *NTHL1* mutations, and impairment of polymerase proofreading due to *POLE* and *POLD1* exonuclease domain mutations [129]. Analysis of CRC organoid mutational signatures was important to validate the association between the most common CRC mutational signature, named SBS44, and MMR gene deficiency, which has been most closely associated with both sporadic and hereditary CRC cases [106,108,132]. More recently, human intestinal organoids exposed to colibactin, a genotoxin compound produced by certain strains of Escherichia Coli bacteria that is significantly increased in CRC patients and involved in tumorigenesis, were characterized by the signatures SBS88 and single deletions at thymine homopolymers called ID18 [74]. Interestingly, other mutational signatures such as SBS17b, dominated by T > G substitutions, were found to be induced in healthy intestinal organoids after 5-fluorouracil (5FU) treatment. This evidence suggests that chemotherapeutic agents may be mutagenic and may induce secondary malignancies [109].

Functional genomic profiling of CRC organoids has also been used as a drug screening tool to identify genotype–drug phenotype correlations [105]. Comparisons between ex vivo CRC organoid responses to anticancer agents and clinical trial outcomes have demonstrated that tumor-derived organoids can accurately mimic patient responses observed in clinical trials, that they represent a powerful tool for implementing personalized medicine approaches and optimizing treatment selection.

Intestinal organoids have also proven to be a valuable tool in the study of inflammatory bowel disease (IBD), a chronic condition that primarily includes ulcerative colitis (UC) and Crohn’s disease (CD) [133,134]. Targeted sequencing of genomic DNA from healthy and CD organoids was performed to analyze approximately 154,000 IBD-associated SNPs. SNPs affecting the bacterial clearance pathway, in particular risk alleles in *NOD2* and *ATG16L1*, as well as CRC-associated mutations, were over-represented in a CD subtype organoid characterized by immune dysregulation that renders the colonic epithelium more susceptible to microbial infection. In contrast, SNPs in genes within a senescence- and DNA damage-induced YAP-IL18 proinflammatory pathway were enriched in the CD subtype organoid characterized by stress and senescence-induced fibrostenosis [110].

Whole genome sequencing has been performed on intestinal organoids derived from patients with cystic fibrosis (CF), providing a valuable tool for studying the disease and evaluating potential therapeutic approaches. CF is caused by mutations in the cystic fibrosis transmembrane conductance regulator (*CFTR*) gene, which encodes an anion channel essential for the normal function of many epithelial tissues. In the apical membrane of the intestinal epithelium, CFTR regulates salt and water transport into the intestinal lumen, a process that can be effectively studied using organoid swelling assays to model CF-related dysfunction, as already reported in the imaging chapter [84].

As observed in other PDOs, CF organoids demonstrated a high degree of reproducibility between mutations found in the organoids and those present in the corresponding patients, supporting the concept that organoid systems reflect the genetic landscape of the original tissues and are a valuable tool for precision medicine. Notably, CF organoids not only captured the genetic diversity of CF mutations, including the 17 most common mutations found in the Dutch CF population, but also harbored 61 rare mutations not previously cataloged in CF databases. This highlights the unique ability of organoids to reveal novel genetic variants and underscores their potential as a powerful tool for studying genetic diversity in CF research [111].

### 3.2. Epigenomics

Epigenetic deregulation has emerged as a paradigm in cancer biology that underlies the hallmarks of cancer cells [135].

Epigenomics of colorectal organoids focuses on the study of genome-wide epigenetic modifications, such as DNA methylation, histone modifications, chromatin accessibility, and non-coding RNA activity, that influence gene expression and cellular behavior. Colorectal organoids derived from healthy and diseased tissues provide a valuable platform for such investigations due to their ability to closely mimic the in vivo tissue architecture and environment. In this context, a recent study investigated the relationship between epigenetic alterations and drug response in CRC organoids using DNA methylation analysis. Methylome signatures divided CRC organoids into two distinct subtypes characterized by the presence or absence of the CpG island methylator phenotype (CIMP) [112]. CRC organoids exhibiting CIMP were characterized by hypermethylation of 50 genes, including *MLH1*, *MGMT*, *BNIP3*, and *CHFR*. This pattern of hypermethylation was highly associated with microsatellite instability and BRAF V600E mutation and was associated with increased sensitivity to several chemotherapeutic agents. This evidence suggests that drug sensitivity may be regulated by epigenetic alterations rather than genetic mutations, and CRC organoids provide a powerful tool for investigating such correlations, offering a model to explore how epigenetic changes, such as DNA methylation patterns, influence therapeutic responses.

Familial adenomatous polyposis (FAP) is an inherited CRC syndrome resulting from germline mutations in the adenomatous polyposis coli (*APC*) gene that is a negative regulator of the Wnt/β-catenin signaling pathway [136]. To better understand the underlying molecular mechanism involved in early tumorigenic events in FAP, organoids derived from FAP-affected patients were subjected to DNA methylation analysis to investigate epigenetic modifications [113]. A total of 358 differentially methylated regions were identified by comparing organoids derived from FAP patients with those derived from healthy individuals. Some of these regions were associated with increased expression of adjacent genes, including F-Box and leucine-rich repeat protein 8 (*FBXL8*) and TRIM31 antisense RNA 1 (*TRIM31-AS1*), which appear to play a role in CRC tumorigenesis. These findings highlight the potential contribution of epigenetic alterations to CRC progression and underscore the importance of DNA methylation profiling in understanding tumorigenesis and identifying therapeutic targets.

Furthermore, epigenetic analysis was successfully performed to better define the mechanisms underlying tumor initiation in Lynch syndrome (LS), an inherited condition that increases the risk of microsatellite instability-high (MSI-H) CRC and is characterized by inactivation of genes involved in the MMR system [137]. In this context, colon organoids from LS patients showed a hypermethylated region of *MSH4*, a defective mismatch repair gene. Interestingly, despite this hypermethylation, *MSH4* expression was found to be elevated, suggesting its potential as a biomarker for LS [114].

Among epigenetically controlled genomic elements, enhancers are attracting considerable attention due to their fundamental role in tumor initiation, progression, and metastasis [138]. A comprehensive study of histone modifications in CRC organoids identified significantly increased chromatin accessibility of YAP/TAZ-specific enhancers as a major alteration in the human CRC enhancerome [112].

Epigenetics has been shown to play a relevant role not only in cancer but also in CD pathogenesis [13]. In particular, genome-wide DNA methylation profiling of organoids derived from CD patients revealed a stable loss of DNA methylation in MHC-I and its transcriptional regulator/transactivator *NLRC5* [116,139]. This loss was associated with increased *MHC-I* and *NLRC5* transcription, which in turn promotes CD8+ T cell activation and contributes to mucosal inflammation, a hallmark of CD [116]. These findings highlight the importance of integrating epigenomics with CD organoids, as this approach allows the elucidation of pathogenic mechanisms linking epigenetic alterations and immune dysregulation, the discovery of potential diagnostic and prognostic biomarkers, and the identification of therapeutic strategies targeting epigenetic mechanisms.

Considering that DNA methylation plays an important role in intestinal development and homeostasis, it was not clear whether epithelial DNA methylation signatures are stable intrinsic cellular properties over time or whether they can be modulated by external factors. In this context, genome-wide DNA methylation profiling of adult, juvenile, and fetal intestinal organoids derived from different segments of the intestinal epithelium (terminal ileum and sigmoid colon) revealed a high concordance with the profiles obtained from their respective primary tissues, as well as the presence of segment-specific signatures. These signatures remained stable over prolonged culture periods, with the exception of organoids derived from fetal material, which underwent methylation changes over time [117].

### 3.3. Transcriptomics

Transcriptomic profiling of colorectal organoids examines the complete set of RNA transcripts to understand the gene expression landscape in different regions of the colon and to identify oncogenic/inflammatory pathways, drug resistance mechanisms, and therapeutic responses in pathological conditions.

In an effort to identify specific CRC signatures, RNA profiling performed on colon organoids derived from healthy individuals and CRC patients revealed the presence of distinct clusters that correspond, although not perfectly, to the histologic subtypes, including adenoma, serrated, MSI CRC, microsatellite-stable (MSS) CRC, and neuroendocrine carcinoma organoids. Each CRC subtype exhibited a unique gene expression profile, with the most dysregulated signaling pathways being PI3K/AKT, Wnt/β-catenin, and TP53. The distinct expression patterns associated with each subtype provided valuable insights into the molecular mechanisms underlying CRC and highlighted potential targets for tailored therapeutic interventions [16,17,101].

Another advancement in the field of transcriptomics is single-cell RNA sequencing (scRNA-seq), which has been used to investigate the cellular diversity of CRC organoids. It was found that CRC organoids are composed of epithelial cells with a variable percentage of stem cell-like cells, depending on the presence of essential stem cell factors such as Wnt-3A and R-spondin [118].

Transcriptomic profiling has also been applied to CRC organoids exposed to chemotherapies and targeted therapies to identify gene expression changes associated with sensitivity or resistance, to highlight predictive biomarkers for patient stratification, and to develop effective therapies for advanced CRC. For example, transcriptomic profiling using RNA-seq identified differentially expressed genes associated with oxaliplatin resistance. Among these, fibroblast growth factor receptor 1 (FGFR1), oxytocin receptor (OXTR), and retinoic acid receptor beta (RARB) are the most relevant as they are targets of FDA-approved drugs [119].

Since drug resistance can also be influenced by the tumor microenvironment (TME), a co-culture model of CRC organoids with cancer-associated fibroblasts (CAFs) was established and transcriptomic profiling was performed after treatment with chemotherapeutic agents to identify resistance-related genes. Differentially expressed genes (DEGs) were identified, particularly those associated with IFNα/β signaling and MHC class II protein complex assembly. These genes play a critical role in modulating the immune response within the TME, thereby influencing tumor response to chemotherapy [120].

While the latter approach investigated how the TME modulates therapeutic responses, other studies focused on identifying cancer-intrinsic molecular signatures that are often masked in primary bulk tumor tissues due to the presence of inflammatory and stromal cells. In this context, RNA-seq of CRC organoids allowed the identification of cancer-intrinsic immunogenic features, in contrast to primary tumor tissues where the immune component of the TME can confound tumor cell-specific signatures. In particular, CRC organoids expressed immune-related genes, including HLA class II genes and genes involved in immune checkpoint regulation [107]. For some of these genes, such as *PD-L1*, there was not always a correlation between expression in CRC organoids and primary tissue, suggesting that such expression in primary tissue is likely related to the resident inflammatory cells. In addition, it was observed that PD-L1 expression in CRC organoids was associated with the alteration of specific signaling pathways. CRC organoids with high PD-L1 expression were enriched in genes associated with interferon α/γ response pathways, whereas those with low PD-L1 expression tended to be enriched in genes related to the Wnt/β-catenin pathway. Notably, HLA-II appeared to be an important intrinsic factor associated with patient survival.

Unfortunately, RNA signatures are rarely reliable predictors of drug response in clinical settings, as transcriptomic profiles often lack correlation with proteomic expression due to significant post-transcriptional regulatory processes. This aspect highlights the limitations of relying solely on transcriptomic data to predict therapeutic outcomes [140].

Therefore, proteotranscriptomic analysis was performed on CRC organoids derived from pretreated patients to explore the mechanisms underlying drug response. Oxaliplatin non-responder CRC organoids showed an enrichment of the t-RNA aminoacylation process and a shift towards dependence on the oxidative phosphorylation pathway. In contrast, an exceptional response to palbociclib was detected in a CRC organoid characterized by activation of MYC and enrichment of the chaperonin T-complex protein Ring Complex [121]. These findings highlight the importance of integrating proteomic and transcriptomic data to uncover the molecular mechanisms driving drug resistance and sensitivity.

Gene expression profiling by RNA sequencing was also performed in organoids derived from CD patients [110]. As mentioned in the genomic section, organoids from CD patients have different gene expression patterns compared to normal organoids and are segregated into different clusters characterized by upregulation or downregulation of differentially expressed genes. In particular, two clusters were identified. The first cluster, named immune-deficient infectious CD (IDICD), showed upregulation of genes involved in intestinal infectious diseases and the butyrophilins and downregulation of genes involved in the major interferon, chemokine, and cytokine signaling pathways. In contrast, the second cluster, named senescence and stress-induced fibrotic CD (S2FCD), showed upregulation of genes involved in oncogene and oxidative stress-induced senescence, cellular response to stress and defects in apoptosis, and downregulation of genes involved in inhibition of fibrogenic transforming growth factor β signaling [110]. As reported in the genomic section, SNPs impairing bacterial clearance were enriched in the IDICD subtype, whereas SNPs associated with senescence-related DNA damage and inflammation were predominantly enriched in the S2FCD subtype. Thus, there was a clear convergence between the transcriptome and the genome on related processes.

A further advance in transcriptomics applied to organoids from CD patients is represented by scRNA-seq analysis of rectal organoids from individuals with perianal fistulizing Crohn’s disease. This study identified four basic epithelial cell lineages: undifferentiated/stem, early absorptive, early secretory, and a fourth group comprising lineages containing a microfold-like cluster, each characterized by the expression of specific markers. It was also observed that the distribution of each cell type and the expression of specific pathways differed between organoids derived from healthy individuals and those derived from patients with perianal fistulizing Crohn’s disease, with or without inflammation. For example, organoids from the inflamed group showed decreased expression of fatty acid and cholesterol metabolic pathways and increased expression of epigenetic pathway genes involved in chromatin modification compared to organoids derived from patients with non-inflamed perianal fistulizing CD or healthy controls [122].

### 3.4. Proteomics

Proteomics of colorectal organoids is an emerging field providing valuable insights into tissue biology, disease mechanisms, and therapeutic responses [141]. Gonneaud A. et al. described for the first time a method based on stable isotope labeling with amino acids in cell culture (SILAC) for quantitative proteomics analysis of intestinal organoids [123]. To determine quantitative changes in protein content, proteomic analysis was performed in intestinal organoids treated with a histone deacetylase (HDAC) inhibitor known to affect intestinal epithelial cell growth and differentiation. Consistent with the reduction in organoid size and the proliferation of crypt budding structures, proteomic analysis revealed an increase in proteins involved in metabolic pathways such as oxidoreduction, carbohydrate catabolism, and complex sugar absorption. In contrast, proteins associated with DNA replication, cell cycle regulation, and chromosome organization were significantly reduced in intestinal organoids after HDAC inhibitor treatment.

In organoids derived from pathological conditions, proteomics identifies dysregulated proteins, such as upregulated oncogenes and altered metabolic enzymes, and elucidates drug resistance mechanisms through proteomic changes [142]. Thus, a proteomic analysis was performed on CRC organoids to identify proteins that could contribute to the generation of a personalized proteome useful for therapeutic strategies. Indeed, significant differences in proteins belonging to the Wnt pathway, which is frequently mutated in CRC, were observed when CRC and healthy organoids were compared [124]. Among the differentially expressed proteins, downregulation of the tumor suppressor gene MYO1C [143] and Desmocollin-2, which is usually decreased or absent in CRC [144], were observed. In contrast, protein overexpression in CRC organoids was observed for ubiquitin-conjugating enzyme E2C (UBE2C), which is reported to play an essential role in cell progression [145], and for HspBP1, a co-chaperone that inhibits the activity of Hsp70 and was also found to be overexpressed in several tumors [146].

Proteomic studies of organoids from IBD patients have not been performed, except for one study in which organoids from a canine form of IBD showed a proteomic profile characterized by upregulation of proteins associated with DNA metabolism and cell junctions and downregulation of adhesion molecules and proteins associated with immune defense processes [125].

### 3.5. Metabolomics

Metabolomic studies of colorectal organoids have focused on metabolic dysregulation commonly associated with diseases such as CRC and ICB to identify potential biomarkers and therapeutic targets. In addition, analysis of how colorectal organoids metabolize drugs helps predict treatment efficacy or toxicity. Mass spectrometry (MS) and nuclear magnetic resonance (NMR) spectroscopy are the two most commonly used methods for metabolic profiling. The former offers high sensitivity but requires extensive sample preparation, while the latter is less sensitive but highly reproducible and does not destroy the sample. Metabolic profiling of CRC organoids using high-resolution magic angle spinning magnetic resonance spectroscopy (HR-MAS MRS), an NMR-based technique, revealed a progressive increase in lactate levels along with a decrease in myo-inositol and phosphocholine levels. These metabolic changes were associated with tumor progression and an accumulation of mutations in key genes, including *APC*, *K-RAS*, *p53*, and *SMAD-4* [126]. Another reason for performing metabolic profiling of CRC organoids is to identify metabolites that could serve as biomarkers of therapeutic response to a specific treatment. In this regard, the metabolic profile of CRC organoids treated with 5-FU, a drug commonly used in CRC patients, revealed modulations in metabolites involved in pyrimidine and purine metabolism as well as lipid metabolism. Specifically, 5-FU treatment induced an increase in 2′-deoxyuridine levels and a decrease in 2′-deoxyadenosine, acylcarnitine, and phosphatidylcholine [127].

An interesting application of metabolic profiling in CRC organoids is the identification of metabolic effects of specific therapeutic approaches. In this regard, CRC organoids were treated with 4-iodo-6-phenylpyrimidine (4-IPP), an inhibitor of the MIF/CD74 signaling axis, which is known to play a role in the regulation of cellular metabolism and energy balance. Metabolic analysis revealed a marked increase in pyruvate levels along with a decrease in key TCA cycle metabolites, attributed to mitochondrial disruption caused by suppression of MIF activity [128]. Furthermore, exposure of CRC organoids to metformin, which inhibits mitochondrial respiratory chain complex I, induced a strong reduction in cholesterol and membrane fatty acids, suggesting that these metabolic changes may be both the consequence and the mechanism responsible for metformin-induced mitochondrial dysfunction [128]. The combination of metabolic profiling in CRC organoids provides a deeper understanding of the cellular impact of targeted treatments and may be useful for identifying novel metabolic biomarkers.

## 4. Organoids as Models for Therapy

Sato et al. pioneered the establishment of primary human CRC organoids that closely mimic the histological features of the tumor of origin, exhibiting patient-specific morphologies and a 94.4% overlap in the NGS mutational spectrum [8]. Several studies have confirmed the good mirroring of organoids with the tumor mutational status and the ability to predict drug response [147,148]. PDOs accurately reflect the genetic heterogeneity and regional characteristics of diseases such as celiac disease [149], IBD [150], and CRC [118]. Thus, organoids are reliable models for developing therapeutic strategies and informing clinical decisions with functional data. Martini et al. derived organoid models from 40 primary CRC or metastases using a 6-day test with most clinically available drugs [151]. This screening not only identified expected drug resistance (i.e., *KRAS* mutation-driven Cetuximab resistance) but also suggested efficient drug combinations for three relapsing, multidrug resistant cases. Using a microfluidic chip, Chen et al. were able to map tumor heterogeneity using a single cancer stem cell culture approach [152]. Single-cell-derived tumor organoids exhibited a distinct genetic background, making them valuable tools for demonstrating tumor heterogeneity as well as conducting personalized therapy-oriented drug screening. This analysis showed that smaller single-cell-derived tumor organoids exhibited higher invasion and drug resistance capabilities compared to the larger ones. As we will see in other chapters in this review, CRC is strongly influenced by its microenvironment, which includes fibroblasts, leukocytes, and endothelial cells. Fibroblasts are enriched in CMS4 CRC and contribute to cancer drug resistance and metastasis. Thus, Farin et al. established a biobank of 30 CRC organoid–fibroblast matched samples and found that fibroblasts strongly influenced organoids’ response to gefitinib [153]. However, the fibroblast protection could be reverted by targeting cMet (HGF receptor).

Organoids have also revolutionized the study of IBD, helping to understand epithelial dynamics and the complex interplay between gut microbiota, immune responses, and genetic susceptibility. By transplanting intestinal organoids into mouse models of colitis, Watanabe et al. demonstrated the ability of organoids to model the pathophysiology of IBD by restoring the epithelium after sodium dextran sulfate (DSS)-induced lesions [154]. This approach opens the way to potential regenerative therapies, such as organoid transplantation in cases of intractable ulcerative colitis. Hou et al. highlighted the interactions between intestinal stem cells (ISCs) and immune cells in organoids and showed that the regeneration of the epithelium after a lesion is closely related to cytokine signaling from immune cell populations [155]. The regenerative capacity of organoids can also be enhanced by specific epigenetic modifiers of the Hippo pathway, such as valproic acid and the EZH2 inhibitor EPZ-6438, which are critical for recovery from inflammatory states [156]. The microbiota also influences intestinal epithelial embryonic development [157] and tissue regeneration in IBD disease [158].

Methodological advances in the generation of lung organoids have aided the understanding of CF. As reported in the imaging chapter, intestinal organoids can be used as a simpler alternative for CF modeling. Spelier et al. established CF patient-derived intestinal organoids with rare mutations, screened 1400 compounds to identify active drugs, and demonstrated that statins can contrast specific CF mutations [159]. While most CF organoid-based studies are limited to drug testing, Bulcaen et al. attempted to correct two CFTR gene mutations (L227R- and N1303K) using prime editing technology in patient-derived rectal organoids [160]. After DNA correction, localization and function of the CFTR protein were restored, confirming that a gene editing therapy approach could be a valuable alternative for future therapies.

### Organoids for Drug Screening and Personalized Medicine

Organoids represent a breakthrough in drug discovery and clinical translation. Numerous studies have identified drugs that can exert direct toxicity or synergize with standard chemotherapy. Among many examples, ML264, a KLF5 inhibitor, improved CRC organoid sensitivity to oxaliplatin [161], AY9944 and GANT61 Hedgehog pathway inhibitors reduced CRC organoid viability in combination with 5-FU or irinotecan [162] and cholesterol biosynthesis inhibitors enhanced the anticancer activity of fasting [163]. Some compounds showed different effects when tested on normal or CRC mucosa. Park et al. discovered that butyrate can be used as a radiosensitizing agent against CRC while protecting normal mucosa and minimizing the associated toxicity [164]. On the contrary, the toxicity test for KAN0438757, an inhibitor of a fructose kinase, showed that this substance selectively stimulated the growth of CRC organoids without affecting normal organoids [165]. These examples strongly support the parallel testing of normal colorectal organoids during high-throughput drug screening for CRC.

The goal of personalized medicine is to provide the most appropriate treatment for each patient. The high degree of morphological, genotypic, and mutational similarity between patient organoids and the original tumor makes organoids the best model for personalized therapy. In the context of personalized medicine, organoids can be used not only to test drugs, but also to monitor changes over time. As a patient receives treatment, organoids can be used to analyze disease response, drug resistance, or adaptation of pathology. This approach provides continuous feedback, allowing therapies to be adjusted in a timely and precise manner. For this reason, PDOs are currently being tested in clinical trials for predictive drug screening (see Table 2). Despite some methodological differences in the evaluation of organoid and patient responses, many studies have reported good concordance. Hsu et al. used a single hit multi-target algorithm to investigate the radiotherapy response of normal mucosa, adenoma, and CRC-derived organoids [166]. According to this algorithm, normal and adenoma-derived organoids were more resistant to radiotherapy compared to CRC. Radio-sensitivity was associated with reduced DNA repair. The comparison of the response of rectal cancer organoids to radiotherapy-treated patients found a close correlation, indicating the potential predictive efficacy of this approach. Janakiraman et al. also found that organoids can reflect the response of matched rectal cancer patients to 5-fluorouracil/radiation (5FU/RT) combination therapy [167]. In this setting, Cetuximab selectively enhanced the effects of radiation in KRAS wt patients. Narashiman et al. used organoids of CRC peritoneal metastases, which typically show strong drug resistance, to identify a drug sensitivity panel for each patient [168]. This approach was able to stabilize the condition of two patients who were unresponsive to standard therapy. Mo et al. found that the IC50 of FOLFOX or FOLFIRI treatment tested on metastatic CRC-derived organoids was lower in patients with stable disease or partial response to treatment, and the opposite was true for patients in progression [169]. Geevimaan et al. found that advanced CRC patients responding to FOLFOX therapy correlated with their matched organoids showing higher sensitivity to oxaliplatin treatment [170]. Despite these examples of good correlation, some studies have reported divergent responses of organoids and patients to the same therapy [171,172]. A recent revision of the literature, comparing organoid and patient response to therapy, found a positive predictive value of 68% and a negative predictive value of 78%, exceeding the response rates observed with an empirical treatment but confirming the incomplete predictive power of organoids [99]. The main variables affecting the predictive power of organoids for personalized drug screening are an insufficient establishment rate of proliferating cultures, the different culture models (matrix embedding, OoC, ALI), and the overall patient status, which does not always allow systemic treatment with highly toxic drugs [173,174,175]. Also, the composition of the organoid medium, which promotes stem cell proliferation (wnt3a, R-spondin, noggin, EGF) and inhibits some apoptotic mechanisms (nicotinamide, N-acetylcysteine, Y27632, PGE2) could influence organoid behavior and resistance to therapy. Cancer organoids are also used to study drug resistance and identify the underlying mechanisms. Resistance evolves over time and involves reversible phenotypic changes, such as senescence processes, metabolic reprogramming, epigenetic modifications, changes in the tumor microenvironment, epithelial–mesenchymal transitions, and/or irreversible mutations [176,177], all of which can be addressed by omics approaches, as discussed in the previous chapter.

## 5. Organoids as Immunotherapy Models

**Figure 5:**
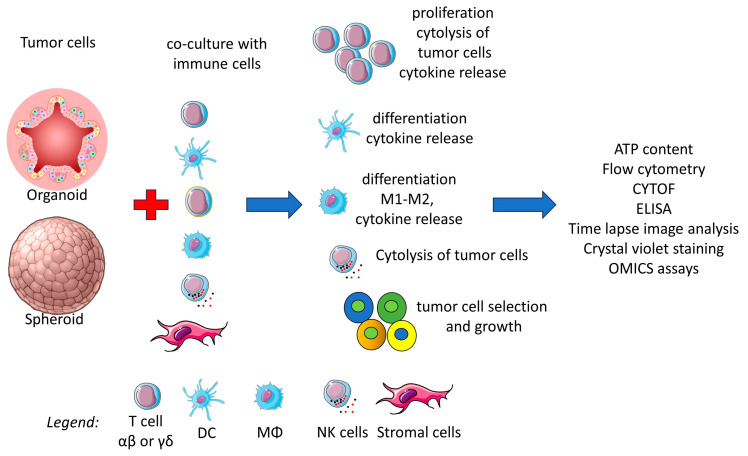
Organoids and spheroids as immunology models. Both tumor cell organoids and spheroids can be used to analyze the adaptive and innate anti-tumor immune responses. Usually, organoids derived from the mucosa of CRC patients or spheroids obtained after culture of either primary or established CRC cell lines are co-cultured with immune cells. Immune cells can be represented by unselected peripheral blood mononuclear cells, peripheral blood T cell subsets, monocytes or monocyte-derived dendritic cells (DC), NK cells, or mesenchymal stromal cells. The immune response can be assessed, and the proliferation, cytokine production, differentiation of cultured leukocyte populations, and anti-tumor properties such as killing activities can be evaluated. Several assays can be applied to study the immune response to 3D CRC cultures, such as the content of ATP, staining with crystal violet after adhesion of 3D cultures, conventional multicolor flow cytometry, or mass flow spectrometry (Cytof). The cultures can be followed by time-lapse imaging, and the resulting cells, either tumors or leukocytes, can be analyzed by OMICS techniques to define the selection of tumor cells and the differentiation of leukocytes in detail. These co-cultures can be used to study the molecular mechanisms involved in tumor cell–leukocyte cross-talk, such as the generation of an antigen specific immune response, NK cell cytotoxicity, and features of tumor cells resistant to immunotherapy.

The innate and adaptive immune responses are a hallmark of cancer [178]. Immune response can play a key role in the clinical outcome of MSI CRC, but it is still uncertain whether it is possible to trigger the host immune response in MSS CRC [179]. The significant phenotypic and molecular heterogeneity of CRC tumors, both among patients and within one patient, also depending on the tumor location, is a major concern [180]. The study of the immune response using established cell lines with specific, or even unique, phenotypic and molecular features does not resemble the in vivo situation. It is plausible that effector lymphocytes encounter various types of epithelial tumor cells forming a cancer tumor mass. CRC cell lines have been and continue to be a valuable tool for studying tumor–immune system interactions due to their consistent response to drugs and immune cells. However, the need for a more reliable model fitting patients’ situations has become more urgent based on the constant increase in therapy-resistant tumor cell clones [181,182,183]. These resistant cells can be either induced or selected by the therapy and are clearly linked to the heterogeneity of the tumor [184]. Tumor cell spheroids and patient-derived CRC organoid cultures can serve as viable in vitro models for studying antitumor immune responses [182,185,186]. These 3D culture conditions facilitate immune–tumor cell interactions that more closely resemble the in vivo situation, compared to the cross-talk observed in conventional 2D cultures. As discussed in the previous chapters, organoids can mirror the variety of CRC epithelial cells, making them a valuable tool for studying personalized therapy. Cell components of innate immunity include several leukocyte subsets such as monocytes and monocyte-derived macrophages, myeloid-derived suppressor cells, and natural killer lymphocytes [187,188]. In contrast, T cells that bear the T cell antigen receptor represent the major effector cell population involved in antitumor adaptive immunity, responding to tumor-specific or tumor-associated antigens. Among lymphocyte populations, innate lymphoid cells (ILCs), mucosal-associated T cells, and T cells bearing the γδT cell receptor (TCR) can display strong antitumor activity and play effector and regulatory roles in CRC cell growth and diffusion [188,189,190]. In this report, we will present a critical analysis of the major findings reported on the responses detected in co-cultures of immune cells and 3D CRC cultures (Figure 5).

### 5.1. Three-Dimensional Co-Cultures of T Lymphocytes and CRC

A panel of CRC organoids can be used to trigger a T cell-mediated immune response against CRC [31]. Based on a series of 15 mismatch repair deficient (dMMR) CRC organoids leading to MSI, it has been shown that co-culture with autologous peripheral blood mononuclear cells (PBMCs) can generate tumor-specific reactivity [31]. This was the first organoid-based demonstration that anti-CRC activity can be obtained from self-peripheral blood T lymphocytes, suggesting that organoids may be a tool for generating in vitro T cell-specific antitumor cells. Co-cultures of PBMCs and organoids were generated using a complex mixture of stimuli (plates coated with an anti-CD28 monoclonal antibody (mAb) in the presence of soluble anti-PD1 mAb and exogenous IL2). IFNγ was added to promote the upregulation of HLA class I antigens. These culture conditions partially bypassed the need for the second signal to trigger T cell proliferation, and the addition of IFNγ maintained the expression of HLA-I, supporting the presentation of CRC organoid-derived antigens in the co-cultures. The addition of IL2 avoided the possible negative regulatory effect of Treg CD4+ T cells present in the PBMCs. It is also conceivable that the monocyte fraction of PBMCs may have played a role as antigen-presenting cells both as monocytes and as dendritic cells [31]. Under these experimental conditions, both T cell activation and tumor cell killing were detected in all co-cultures of CRC organoids and PBMCs. However, the response was minimal (less than 5% of the total CD8+ T cells present in the co-cultures), except for one organoid derived from CRC lymph node metastasis showing 50% activated CD8+ T cells. No T cell response was observed in co-cultures of PBMC with organoids derived from healthy colon mucosa. Taken together, these results indicate that an immune response can be induced in dMMR CRC using peripheral blood T cells and that this response is stronger in CRC lymph node metastases. Three weekly stimulations with CRC organoid cells should enhance the T cell response and the expansion of specific T cells. The low level of response observed may be attributed to the use of unselected PBMC as well as the excess IL2 (150 IU/mL) and IFNγ (200 ng/mL) present in the culture. In addition, half of the culture medium was refreshed with IL2 and anti-PD1 mAb two to three times per week. Finally, the CRC cells forming the organoids were dissociated and cultured in a medium designed for lymphocytes. This procedure could affect the vitality of the CRC cells and generate numerous non-specific signals for PBMCs or T cells. These signals may be caused by danger-associated molecular patterns (DAMPs), which may interfere with optimal T-cell growth. The increase in CD107a expression and intracellular positivity for IFNγ in CD8+ T cells, together with the labeling of tumor cells with an appropriate probe, indicate that T cells can activate effector functions and perforin release upon interaction with CRC organoids.

Further experimental evidence has confirmed that CRC organoids serve as a suitable platform for testing the reactivity of tumor infiltrating lymphocytes (TILs) directed toward tumor neoantigens [191]. Immunological assays of TILs have shown that CRC organoids can be used to identify differences among TCRs recognizing the same antigen and to select TCRs that react with private neoantigens. TIL-CRC organoid models can identify tumor-specific defects that impair TIL responses. These findings relate to CRC organoids derived from metastases (mostly liver or lung, see Supplementary Information of Parikh et al. [191]). However, the molecular and phenotypic characterization of these organoids was not detailed (e.g., no mention of the stage, grade, and the MSI or MSS status), preventing comparison with those described in other reports [179]. In conclusion, it is conceivable that human CRC organoids may be useful on an individual patient basis to help identify the key factors involved in an efficient anti-tumor immune response.

MSS CRC, characterized by a low tumor mutation burden (TMB) [179], has fewer tumor neoantigens compared to MSI CRC, preventing efficient antigen-specific immune responses. In a murine model, using the orthotopic implantation of human CRC organoids into the distal colon of mice, it was demonstrated that the low expression of neoantigens in MSS CRC impairs effective cross-priming, leading to T cell dysfunction [192]. Remarkably, the therapeutic or experimental reconstitution of this priming allowed an efficient T cell response, even with low neoantigen expression. In this study, all human MSS CRC tumors expressed at least two clonal predicted neoantigens, but the expression was significantly lower compared to MSI patients. The development of a fully engineered murine organoid model carrying the key gene mutations of human metastatic MSS CRC [193], such as APC, KRAS, TP53, and SMAD4, allowed the study of T cell-mediated immune responses. Importantly, this organoid model, besides expressing the reported genetic alterations, expressed the CD8+ T cell antigen epitope SIINFEKL directly linked to Apc knockdown; the expression of this epitope could be markedly modulated (from basal level to 400-fold increase) in the context of MHC. Mouse models with the highest and intermediate expressions of the SIINFEKL epitope induced efficient tumor elimination, while the organoids with the lowest expression did not. This finding suggests the possibility of generating an efficient immune response in MSS CRC if the antigen epitope is abundantly expressed. Tumor-specific neoantigens were present in all organoids regardless of the expression of the reported epitope and tumor infiltration with CD8+ T cells. In tumors with low response, there was a dysfunctional T cell response with reduced magnitude, TCR diversity, and cell functionality. Moreover, these T cells expressed high levels of regulatory molecules such as PD1, TIM3, LAG3, and 2B4, but low levels of TCF1 and GRZB. The priming of dendritic cells (DC) is the key step to obtaining a response. In the organoid model with low expression of the SIINFEKL epitope, treatment with anti-CD40 antibody either alone or in combination with anti-immune checkpoint antibodies (anti-PD1, anti-CTLA4, or anti-TIM3) could reactivate an anti-tumor response. CD40/CD40 ligand interaction induces DC differentiation, and differentiated DCs can efficiently help the T cell-mediated immune response [194]. Notably, the use of anti-immune checkpoint antibody alone did not induce T cell-specific responses, mirroring MSS CRC. The priming of antigen-presenting cells is thus essential to elicit an appropriate response in this MSS CRC model [193].

Mouse engineered organoids are suitable animal models but may not represent the clonal heterogeneity in human tumors. Multiple neoantigens from different clones may be present in human CRC, and the immune response in mice and humans may involve different components of the TME. In addition, some components may carry different activating or inhibitory receptors [195,196]. In vitro-cultured organoids are composed of both differentiated and stem epithelial cells, but not stromal cells and TILs found in the TME; however, an ALI model, based on whole colon tissue, allows the modeling of human and mouse CRC 3D cultures composed of tumor epithelia, fibroblasts, and infiltrating immune cells (T, NK, NKT cells, and macrophages). These cultures showed gene expression and immune TCR repertoire of the original tumors. In these models, it was possible to expand TILs with specific tumor cytotoxicity by blocking PD1 and/or PDL1 [197]. These ALI cultures have a limited lifespan, and some cell populations (e.g., fibroblasts) deplete quite rapidly, although several phenotypic and functional assays can be performed. Using exogenous IL2 (100–6000 IU/mL depending on the donor), the time culture limit of TILs was approximately 60 days. Cultures derived from murine cell lines were easier to maintain and more reproducible than those obtained from human samples.

In a different approach, a co-culture model of patient-derived primary CRC spheroids and autologous tumor-associated lymphocytes was used to demonstrate the relevance of CD39 antigen blockade [198]. CD39 is a surface ectonucleoside triphosphate diphosphohydrolase-1 [199,200] that hydrolyzes ATP, ADP, UTP, and UDP. CD39 in conjunction with CD73 can lead to the production of adenosine. Adenosine in turn can downregulate the immune response. The expression of CD39 and PD1 on TILs was increased compared with T cells from normal mucosa. The blockade of CD39 with specific antibodies could increase autologous T cell infiltration and the destruction of tumor CRC spheroids [198]. It should be noted that these tumor spheroids were different from conventional organoids. CRC cell suspensions were cultured in 2D in 10% FCS-containing medium for up to 10 days [201]. Afterwards, they were trypsinized and cultured in ultra-low-adherent 96-well-plates to generate spheroids. These cultures contained 12.5% EpCAM-positive cells, thus the spheroids could be composed mainly of fibroblasts, according to the 2D image morphology. Overall, the above reported findings strongly support the notion that 3D cultures of organoids, orthotopically transplanted organoids, AIL CRC cultures, and mixed tumor primary spheroids can be used to analyze the response of the patient to conventional and novel immunotherapies based on the regulation of the specific T cell-mediated immune response.

It is important to note that the frequency of antigen-specific T lymphocytes present in the TME is quite low [202,203]. Therefore, 3D CRC cultures could be a tool for detecting whether other TIL populations, such as innate lymphoid cells (ILCs), mucosal-associated T cells, and T cells bearing the γδ TCR, may exert an antitumor effect [195,196,204,205]. The γδ T cells represent a lymphocyte subset with a potent anti-tumor lytic activity present in the gut as intraepithelial cells. Several types of γδ TCR T cells have been detected in the colon displaying anti-tumor or even pro-tumor activities [204,205,206,207,208,209]. Among these γδ T cell subsets, it has been demonstrated that Vδ2 T cells of CRC specimens can be expanded with either soluble or nanoparticle-carried aminobisphosphonates such as zoledronic acid [206]. More relevant, these Vδ2 T cells can efficiently kill CRC organoids. This finding also suggests that Vδ2 T cells are good antitumor effector cells, like specific αβ T cells [31,197]. In addition, Vδ2 T cells from autologous peripheral blood co-cultured with CRC organoids could proliferate in response to the therapeutic anti-epidermal growth factor receptor (EGFR) antibody Cetuximab conjugated with zoledronate (Cet-ZA). This stimulation is consequent on the interaction and endocytosis of the Cet-ZA/EGFR complex and production of small pyrophosphate antigens such as isopentenyl pyrophosphate (IPP). It has been reported that IPP can be presented to the Vδ2 T cells by members of the butyrophilin family such as BTN3A1 and BTN2A1 [210,211,212,213]. CRC organoids are positive for both BTN3A1 and BTN2A1 molecules. Vδ2 T cells could also kill CRC organoids by antibody dependent cytotoxicity (ADCC) and Vδ2 TCR-mediated engagement. This effect was evident both in an autologous or allogeneic setting and using organoids from either MSI or MSS CRC. Altogether, these data indicate that Vδ2 T cells are good anti-tumor effector cells and that CRC organoids can be used as a platform to study the molecular mechanisms by which novel conjugated antibody–drugs like Cet-ZA can trigger Vδ2 T cell functions. The possibility of showing a cytotoxic effect under allogeneic conditions would suggest the possibility of using allogeneic Vδ2 T cells for adoptive immunotherapy [209]. In general, γδ T cells, including Vδ2 T cells, do not classically recognize the antigen within the context of MHC-I [210]. However, expanding Vδ2 T cells requires the addition of exogenous IL2, usually at a lower concentration (30 IU/mL) than that used for the generation of neoantigen-specific T cells [31,197]. Therefore, the reported stimulation with Cet-ZA ADC could not be translated in a clinical setting without the addition of IL2. While low doses of IL2 are used in the clinic to activate Vδ2 T cells [214], IL2 can elicit undesired life-threatening side effects [215]. A reasonable solution could be the generation of fusion Ab-IL2-ZA ADCs.

### 5.2. Interactions Between Antigen-Presenting Cells and CRC Organoids

CRC organoids can be used to study the molecular mechanisms and therapeutic targets of innate cells [185]. Among innate cells, antigen-presenting cells (APCs) such as monocytes/macrophages and DCs play a key role in the antitumor immune response [216]. Indeed, DCs can produce cytokines involved in the activation and differentiation of NK cells, as well as present peptide antigens to T cells to trigger antigen recognition [217]. It has been reported that the phenotype and functional features of peripheral blood monocyte-derived DCs could be locked in a more immature state associated with tolerance and pro-tumorigenic effects [218,219,220,221]. DCs were used in co-culture with CRC organoids as immature DCs (iDCs) or after differentiation to mature DCs (mDCs). GM-CSF and IL4 were used to generate iDCs from monocytes, while a combination of IL6, IL1β, TNFα, and PGE2 was required for mDC [222]. Two CRC liver metastasis organoids were used in the co-culture experiments: one with a cystic morphology and a second with a dense appearance under microscopic analysis. These organoids induced different behaviors, distributions, activation, and functions in DCs. DCs penetrated better into the cystic organoid, while the dense organoid was more efficient at inhibiting allogeneic T cell proliferation induced by both iDCs and mDCs compared to the cystic organoid. This effect was mediated by reduced expression of CD86 and HLA-DR and upregulation of PD-L1. These results suggest that CRC organoids can influence DC function and impair allogeneic antigen presentation [222]. It remains to be defined whether the different characteristics of cystic or dense organoids are typical of these two CRC liver metastasis organoids or are common to all CRC organoids with these morphological characteristics. Recently, it has been shown that conventional DC type 2 (DC2) relevant for the induction of T helper cell responses [223] can shift to a CD14+ population with impaired T cell-activating capabilities when co-cultured with CRC organoids [224]. These populations were similar to the DC3 subset, enriched in the peripheral blood of metastatic CRC patients [221,225]. Generation of a DC3-like subset can be achieved by culturing DC2 with IL-6, PGE_2_, and M-CSF as well as tumor cell supernatant [220,226]. The observed shift to the DC3 subset obtained in DC2-CRC co-cultures was partially reduced by blocking PGE2 and IL6, two factors commonly released by CRC cells. This finding supports that this 3D culture model could be used to study therapies aimed at mitigating tumor-induced effects on DCs. The frequency of DC3-like cells varied depending on the CRC organoid used. This could be related to the genotypic and phenotypic landscape of the organoid used. These features were not reported in detail, although different secretomes could be detected among the four organoid-DC2 co-cultures tested [222].

The results reported with organoid–DC co-cultures suggest that the TME can strongly influence the phenotypic and functional behavior of innate cells. However, an organoid is far from recapitulating the TME of CRC. Co-cultures of carcinoma-associated fibroblasts (CAFs), monocytes, and CRC organoids can induce the partial differentiation of monocytes into macrophages, characterized by the expression of some relevant markers typical of tumor-associated macrophages (TAMs) [227]. Indeed, the triple co-culture of CRC organoids and CAFs with monocytes from healthy volunteers led to monocyte differentiation and activation. Specifically, monocytes upregulated mRNA encoding the activation markers CD14, FCN1, and CD86, as well as receptors associated with M2 immunosuppressive macrophages such as MRC1 (encoding CD206) and CD163. Flow cytometric analysis revealed that the coculture of monocytes and CAFs could upregulate surface markers, including CD206, CD163, PDL1, 4-1BBL, and CD86, while SIRPα was downregulated. Conditioned medium from CAFs did not induce the same effects and the addition of organoids did not alter this phenotype, except for the increase in PDL1. The standard chemotherapy for CRC, consisting of oxaliplatin and 5-fluorouracil, induced the polarization of macrophages to a pro-inflammatory phenotype in this model, increasing the mRNA for inflammatory cytokines, such as IL1β1, IL6, and TNFa, and increasing the phagocytosis of tumor cells. Collectively, these results indicate that a triple co-culture system can mimic the in vivo TME and recapitulate some of the events following chemotherapeutic intervention [227].

### 5.3. Innate Cytolytic Cells Can Affect the Viability and Growth of CRC Organoids

It is well known that lymphokine-activated killer (LAK) cells are potent antitumor effector lymphocytes [228]. LAK cells represented by NKT-like cells (mainly CD3+ CD16+ CD56+ CD8+) [229] were obtained by culturing PBMCs with a high dose of IL2 (100–1000 IU/mL) for a short time (48–72 h). LAK cells expressing high levels of the immune checkpoint receptor PD1 on a subset of cells (about 10%) were co-cultured with CRC organoids from MSS+ CRC patients either before or after treatment with IFNγ or a peroxisome proliferator-activated receptor-gamma (PPARγ) agonist [230]. PPARγ can be induced by antidiabetic drugs, including rosiglitazone or pioglitazone [231], and triggers the upregulation of PDL1 in MSS tumor organoids [231]. It has been reported that the upregulation of PDL1 on CRC cells, induced by PPARγ or IFNγ, can favor the triggering of ADCC by LAK cells when treated with the anti-PDL1 antibody atezolizumab. This would suggest that the repurposing of anti-diabetic drugs and biologics such as IFNγ may enhance the antibody–target interaction favoring ADCC, allowing a reduced dose of therapeutic antibodies, thereby lowering costs and side effects. This therapeutic approach could specifically target MSS CRC, as all CRC MSS organoids analyzed showed reduced proliferation and vitality upon treatment with the combination described above. However, this model used high doses of anti-diabetic drugs, IFNγ, and anti-PDL1, which could not be paralleled in patients.

Efficient targeting of CRC organoids has been reported using chimeric antigen receptor (CAR)-engineered NK-92 cells [232]. NK-92 cells are IL2-dependent NK cells derived from the PBMCs of an adult male with non-Hodgkin’s lymphoma [233]. This cell line does not resemble the typical CD16+ CD56low peripheral NK cells as it expresses high levels of CD56 but is CD16-, and it can efficiently kill the NK cell target K562. Organoid targeting was studied with NK-92 cells expressing CAR for the epithelial cell adhesion molecule (EPCAM), the neoantigen EGFRvIII found in several cancers, or the Frizzled receptor upregulated in some CRC patients [232]. Cytotoxic experiments had to be performed in the absence of nicotinamide, as this additive of the organoid medium markedly inhibited NK-92 cytotoxicity. NK-92 cells were tested against 3D organoids embedded in Matrigel or against organoids plated on Matrigel. NK-92 cells were more active against 2D cultures, migrating into and killing CRC organoids while failing to penetrate the matrix. Conceivably, the use of NK-92 CAR cells could be efficient against any type of CRC, either MSI or MSS, if the antigen is expressed on CRC cells. Of course, to avoid side effects induced by NK-92 CAR cells, their specificity should be directed to antigens or neoantigens that are expressed and/or upregulated on tumor cells. This is the case for the neoantigen EGFRvIII and frizzled receptors, which are present on a fraction of CRC.

## 6. Biobanks in the Era of Translational and Personalized Medicine

Biobanks play a crucial role in the collection, processing, storage, and redistribution of biological samples to provide high quality biospecimens and associated data for translational medicine efforts aimed at closing the paradigm “from the patient’s bed to the laboratory bench, and back to the patient” [234]. Reliable biospecimens are a pivotal requirement to ultimately confirm and validate both basic and pre-clinical research. A deeper understanding of the biology of diseases drives biomarker discovery and further validation to exploit their clinical utility according to a personalized approach to treat cancer [235].

The large and complex heterogeneity of biobanks calls for the standardization of their framework and for process harmonization to foster future research data reliability and reproducibility [236]. Despite differences in ‘mission’, sample origin, and type, or whether they are population-based or disease-oriented, biobanks for non-renewable biospecimens can rely on guidelines, international standards, governing bodies, and national and international infrastructures [237]. A quality management system (QMS) regulates and oversees daily biobanking procedures, operator continuous training, corrective actions for non-conformities, personnel safety, and instrument maintenance [238]. Biobanks deal with ethical, regulatory, and privacy issues in a time and age when blurred lines between ethical principles, research, and clinical care are frequently difficult to sharpen [239,240,241].

### 6.1. Living Biobanks for Organoids: Challenges for Standardization

With their unparalleled potential for facilitating functional experiments, organoids greatly surpass traditional pre-clinical models such as 2D cell cultures of immortalized cell lines, patient-derived tumor xenografts (PDTXs), and animal testing [242]. In contrast to the non-renewable biospecimens found in traditional biobanks, organoids are living biomaterials that can be continuously cultured and manipulated.

In recent years, living biobanks of organoids have grown exponentially within the infrastructures of academic institutions and have also been established by commercial entities as part of next-generation biobanking endeavors [243,244].

The Netherlands, U.S., and China have the highest number of biobanks. The majority contain mostly digestive and genital system-derived organoids. Colorectal cancer, pancreatic cancer, breast cancer, glioma, and bladder cancer are the most established tumors [245].

Living biobanks of organoids are responsible for initial culture set-up and expansion, storage procedures, and quality checks to ensure that organoids retain the original tumor characteristics [246,247]. Additionally, when applicable, they provide matched normal organoids from large numbers of individuals.

Despite the advent of intestinal organoid culture technology almost 15 years ago, the (pre-) clinical implementation of organoids is still hampered by non-standardized procedures and ill-defined protocols [248].

It is therefore essential to establish comprehensive guidelines and harmonized protocols that include rigorous quality control checkpoints. The envisioned framework is anticipated to be more intricate and detailed than the ISOs and best practices currently in place for biobanking nonrenewable biospecimens. Standardization aims to ensure consistency and reproducibility in the development, characterization, and result interpretation, resulting in an overall improved success rate. Lee H et al. recently proposed a comprehensive blueprint for proper terminology nomenclature and stepwise approach for protocol standardization and rigorous quality control encompassing organoid characterization, requirements, and post-storage evaluations throughout all stages of development [249].

### 6.2. Living Biobanks for Organoids: Ethical, Legal, and Social Issues (ELSIs)

Organoids’ remarkable impact on basic and clinical research and potential healthcare market necessitates an in-depth re-evaluation and reshaping of the ethical and social issues associated with their use to ensure the rights and privacy of donors are protected [250,251,252,253].

Organoid biobanking raises specific ethical and practical challenges requiring a paradigm shift in science and society within a Responsible Research and Innovation Framework and rethinking concepts and practices related to consent, commercial access, and commodification, privacy, and ownership [254,255].

As more than a participant, being a partner is critical for biobank research on organoids for translational and precision medicine purposes. First, the specific characteristics of future research are unknown at the time of consenting for inclusion in the biobank; therefore, a model of participatory consent over time and participatory governance (see an example in https://repository.bbmri.it/s/stC8Lc4kPDn2qQt accessed on 13 March 2025) to ensure adjustment to changing ethical requirements [256] is a cornerstone that aims for responsible biobank research through the establishment of a continuous relationship between the biobank and participants in complex tissue biobanking translational medicine. Examples include the involvement of patient organizations in biobank access bodies, participation in advisory board meetings, or consultation rounds to assess decisions or results.

Beyond donation, biobanking should be recognized as a collaborative process and a participatory pact between all the actors involved based on transparency, inclusion, participation, and reciprocal engagement. Each participant is an active and irreplaceable part of the collaborative ecosystem, and in a reciprocal way is necessary for the development of the innovative scientific model required by biobanking and data-based science (see an example in https://repository.bbmri.it/s/stC8Lc4kPDn2qQt accessed on 13 March 2025).

In addition, organoids have a strong social impact and hold significant economic value, leading to strong commercial interests. The ethical challenge is ensuring that the benefits are distributed fairly among all the players [257].

Organoids are three-dimensional structures developed in vitro from stem cells, including human pluripotent and adult stem or progenitor cells [251]. However, there are currently no specific regulations governing research with organoids or their biobanking; therefore, the general rules pertaining to biological materials from which organoids are derived, as well as the overarching regulations related to biobanking and biobank-based research, should be applied. While there is no outright ban on experimentation with embryonic stem cells in the European Union, individual Member States have considerable discretion in this matter. However, research using embryonic stem cells is excluded from funding. This exclusion is reinforced by Article 19 of Regulation (EU) 2021/695 (http://data.europa.eu/eli/reg/2021/695/2024-03-01 accessed on 13 March 2025), established by the European Parliament and the Council on 28 April 2021, which governs Horizon Europe: the Framework Programme for Research and Innovation. This regulation lays down the rules for participation and dissemination while repealing previous Regulations (EU) No 1290/2013 and (EU) No 1291/2013 (http://data.europa.eu/eli/reg/2013/1291/oj accessed on 13 March 2025). Regarding adult stem cells and induced pluripotent stem cells, the “Guidelines for Stem Cell Research and Clinical Translation” (https://www.isscr.org/guidelines accessed on 13 March 2025) can provide a useful framework. Specifically, in relation to organoids, the Horizon 2020 Project “Hybrida—Embedding a Comprehensive Ethical Dimension to Organoid-Based Research and Related Technologies” (https://hybrida-project.eu/ accessed on 13 March 2025) aims to develop a comprehensive regulatory framework for organoid research and associated technologies.

## 7. Conclusions

Life sciences research has been revolutionized by organoid culture techniques over the last fourteen years. Starting from PSCs or adult stem cells, our ability to propagate normal and pathological epithelia from different human organs is rapidly increasing, allowing the establishment of in vitro models that mimic specialized tissues. Many of the models reported in this review represent future directions for organoid development. Reconstitution of normal tissues is particularly interesting for the study of physiology at the cellular level, monitoring trans-epithelial transport, hormone secretion, digestion/absorption, and infection. On the other hand, a multidisciplinary collaboration between tissue engineers and biologists could lead to significant advances in homologous tissue generation and transplantation, with possible gene therapy approaches against hereditary diseases. Indeed, a diseased organoid that cannot be reconverted to its physiological behavior (e.g., cancer) still represents a unique tool for drug testing. High-throughput assays accompanied by AI-supported analysis could better identify not only cytotoxic effects but also subtle modulation in cell behaviors, e.g., modified stem/differentiated cell polarization. The increasing complexity of in vitro assembloids points to the attempt to reconstitute tissues with cells of different origins. This approach is difficult and, in fact, results in short-lived tissues due to the different in vitro conditions required by different cells to proliferate, and the tendency of one cell population to outgrow others. However, human multicellular assembloids can recapitulate the complex architecture of real tissues, allowing focused studies under ex vivo conditions. A particularly interesting application is immunology, allowing the creation of a full-autologous experimental system. The parallel development of organ-on-chips, air–liquid interface, and tissue printing techniques are contributing to the establishment of more organized and stable tissues that better represent the tissue of origin for long-term tests. Like all pioneering fields, organoid cultures are generating a growing number of different protocols and experimental/technical conditions. This creative approach urgently needs to be flanked by increased standardization, allowing the production of comparable and reproducible results. This standardization necessitates a deeper understanding of the key factors modulating organoids’ behavior, starting from their complex culture medium containing several modulators of defined signaling pathways, matrix embedding (where mouse-derived Matrigel is a great limit for immunology studies), and the definition of experimental endpoints distinguishing short- and long-term effects. The use of human-derived tissues/cells also has important ethical implications. While each patient/donor is the sole owner of his/her own tissue, the procedure for obtaining organoid cultures, which are limited to the laboratory of origin and should be destroyed at the end of the study, represents a major limitation, a waste of resources, and the impossibility of repeating previous tests in the same reference model. In the coming years, we need to unify all these open questions so that we can bring the myriad streams of our research back to the bed of a larger river.

## Figures and Tables

**Table 1:** Omics analyses of healthy and patient-derived colorectal organoids.

Omics Type	Sample Type	Technique Used	Key Findings	Reference
Genomics	CRC organoids	WGS	Comparison of mutational profiling between organoids and parental tumor Mutations and drug response prediction	[105]
Genomics	CRC organoids	WES	Comparison of mutational profiling between organoids and parental tumor	[101,106]
Genomics	CRC organoids	WES	Genomic characterization	[17]
Genomics	CRC organoids	Targeted sequencing	Genomic characterization	[107]
Genomics	CRC organoids	Targeted sequencing WES Comparative genomic hybridization/SNV microarray	Mutational profiling Copy number alterations	[16]
Genomics	CRC organoids	WGS	Origin of SBS44 mutation signature	[108]
Genomics	CRC organoids	WGS	SBS88 mutation signature induced by colibactin	[74]
Genomics	Healthy organoids	WGS	SBS17b mutational signature induced by chemotherapy	[109]
Genomics	CD organoids	Targeted sequencing	SNP identification	[110]
Genomics	CF organoids	WGS	Identification of common and rare mutations of CF	[111]
Epigenomics	CRC organoids	Bisulfite conversion microarray	Association between methylator phenotypes and drug sensitivity	[112]
Epigenomics	FAP organoids	Bisulfite conversion microarray	Identification of differentially methylated regions and association with CRC development	[113]
Epigenomics	LS organoids	Bisulfite conversion microarray	Identification of a hypermethylated region of MSH4 gene proposed as biomarker of LS	[114]
Epigenomics	CRC organoids	ChIP-seq	Histone modifications and increased chromatin accessibility of specific enhancers	[115]
Epigenomics	CD organoids	Bisulfite conversion microarray	Loss of DNA methylation in MHC-I and in its transcriptional transactivator NLRC5	[116]
Epigenomics	Healthy organoids	Bisulfite conversion microarray	Dynamic changes in DNA methylation of different intestinal segments during development	[117]
Transcriptomics	CRC organoids	Microarray analysis	Gene expression profiling of different CRC subtypes	[16]
Transcriptomics	CRC organoids	Microarray analysis	CRC subtype classification based on transcriptomics profiling	[17]
Transcriptomics	CRC organoids	RNA-seq	Transcriptomics profiling and drug response signatures	[106]
Transcriptomics	CRC organoids	scRNA-seq	Comparison of transcriptomics profiling between organoids and parental tumor	[118]
Transcriptomics	CRC organoids	RNA-seq	Identification of drug targets by transcriptomics profiling	[119]
Transcriptomics	CRC organoids	RNA-seq	Expression of immune-related genes	[107]
Transcriptomics	CRC organoids	RNA-seq	Identification of biomarkers associated with anticancer drug resistance	[120]
Transcriptomics	CRC organoids	RNA-seq	Proteotranscriptomics profiling and drug response	[121]
Transcriptomics	CD organoids	RNA-seq	Identification of different clusters through gene expression profiling	[110]
Transcriptomics	CD organoids	scRNA-seq	Gene expression profiling depending on the presence or absence of inflammation	[122]
Proteomics	Healthy organoids	SILAC/MS	Protein expression profiles after drug treatment	[123]
Proteomics	CRC organoids	Nano-UHPLC/MS	Identification of personalized proteomics profiles	[124]
Proteomics	IBD organoids	LC/MS	Characterization of proteomics profiling	[125]
Metabolomics	CRC organoids	HR-MAS MRS	Metabolic alterations associated with tumor progression	[126]
Metabolomics	CRC organoids	LC-QTOF-MS	Characterization of metabolic drug response	[127]
Metabolomics	CRC organoids	GC/MS	Metabolic changes after drug treatment	[128]

Abbreviations: whole genome sequencing: WGS; exome genome sequencing: WES; colon carcinoma: CRC; single nucleotide variant: SNV; Crohn’s disease: CD; cystic fibrosis: CF; single nucleotide polymorphisms: SNPs; familial adenomatous polyposis: FAP; Lynch syndrome: LS; genome-wide chromatin immunoprecipitation sequencing: ChIP-seq; single-cell RNA sequencing: scRNA-seq; stable isotope labeling with amino acids in cell culture: SILAC; nanoliter-ultra high performance liquid chromatography: nano-UHPLC; mass spectrometry: MS; intestinal bowel disease: IBD; high-resolution magic angle spinning magnetic resonance spectroscopy: HR-MAS MRS; liquid chromatography quadrupole time-of-flight mass spectrometry: LC-QTOF-MS; isotope-dilution gas chromatography-mass spectrometry: GC-MS.

**Table 2:** Clinical trials using organoids for therapeutic decision making.

NCT Number	Study Title	Study Status	Results	Conditions	Study Type	Locations
NCT05832398	Precision Chemotherapy Based on Organoid Drug Sensitivity for Colorectal Cancer	RECRUITING	NO	CRC	INT	Guangzhou (CN)
NCT05384184	Next Generation Pre-clinical Model for Colorectal Cancer Metastases and Hepatocellular Carcinomas (BORG)	COMPLETED	NO	CRCm; HCC	OBS	Marseille (FR)
NCT05304741	The Culture of Advanced/Recurrent/Metastatic Colorectal Cancer Organoids and Drug Screening	RECRUITING	NO	CRC	OBS	Chongqing (CN)
NCT06100016	A Clinical Study Aims to Assess the Consistency of Clinical Efficacy in Colorectal Cancer Treatment and Drug Susceptibility Outcomes Using a Novel Drug Susceptibility Testing Method	RECRUITING	NO	CRC	OBS	Shenyang (CN)
NCT05401318	Tailoring Treatment in Colorectal Cancer	RECRUITING	NO	CRN	OBS	Viken (NO)
NCT05267912	Prospective Multicenter Study Evaluating Feasibility and Efficacy of Tumor Organoid-based Precision Medicine in Patients With Advanced Refractory Cancers	ACTIVE, NOT RECRUITING	NO	APST	INT	Villejuif (FR)
NCT05725200	Study to Investigate Outcome of Individualized Treatment in Patients With Metastatic Colorectal Cancer	RECRUITING	NO	CRCm	INT	Oslo (NO)
NCT05038358	Tumor Immune Microenvironment Involvement in Colorectal Cancer Chemoresistance Mechanisms	RECRUITING	NO	CRC	OBS	Grenoble (FR)
NCT06136949	The Theranostic Value of STARD3 in Colorectal Cancer: The STAR Study	RECRUITING	NO	CRC	OBS	Aviano (IT)
NCT04896684	Chronic Intestinal Pathologies Analytical Cohort at TouLouse	RECRUITING	NO	IBD; CRC	OBS	Toulouse (FR)
NCT02732860	Personalized Patient Derived Xenograft (pPDX) Modeling to Test Drug Response in Matching Host	RECRUITING	NO	CRN/CRC; BN/BC; ON/OC	OBS	Toronto (CA)
NCT06349590	Manipulation of the Gut Microbiome by a Standardized Preoperative Diet to Prevent Colorectal Cancer Recurrence and Metastasis Following Surgery	RECRUITING	NO	CRC	INT	Chicago (US)
NCT04622423	Advanced Therapies for Liver Metastases	RECRUITING	NO	PDAC CRC (LM)	OBS	Milan (IT)
NCT04587128	Early-Line Anti-EGFR Therapy to Facilitate Retreatment for Select Patients With mCRC	RECRUITING	NO	CRCm	INT	Madison (US)

Abbreviations: CRN = colorectal neoplasms; CRC = colorectal cancer; CRCm = metastatic CRC; LM = liver metastasis; IBD = inflammatory bowel disease; HCC = hepatocellular carcinoma; BN = breast neoplasm; BC = breast cancer; ON = ovarian neoplasm; OC = ovarian cancer; PDAC = pancreatic ductal adenocarcinoma; APSTs = advanced, pretreated solid tumors; OBS = Observational; INT = interventional.

## Data Availability

Not applicable.
